# MCT4 and CD147 colocalize with MMP14 in invadopodia and support matrix degradation and invasion by breast cancer cells

**DOI:** 10.1242/jcs.261608

**Published:** 2024-04-30

**Authors:** Signe Meng, Ester E. Sørensen, Muthulakshmi Ponniah, Jeppe Thorlacius-Ussing, Roxane Crouigneau, Tanja Larsen, Magnus T. Borre, Nicholas Willumsen, Mette Flinck, Stine F. Pedersen

**Affiliations:** ^1^ Section for Cell Biology and Physiology, Department of Biology, Faculty of Science, University of Copenhagen, 2100 Copenhagen, Denmark; ^2^ Nordic Bioscience A/S, 2730 Herlev, Denmark

**Keywords:** Monocarboxylate transporter, SLC16A3, Basigin, MDA-MB-231, Lactate, Cell migration, Collagen-I, Cortactin, Large acidic vesicles, Invadopodia

## Abstract

Expression levels of the lactate–H^+^ cotransporter MCT4 (also known as SLC16A3) and its chaperone CD147 (also known as basigin) are upregulated in breast cancers, correlating with decreased patient survival. Here, we test the hypothesis that MCT4 and CD147 favor breast cancer invasion through interdependent effects on extracellular matrix (ECM) degradation. MCT4 and CD147 expression and membrane localization were found to be strongly reciprocally interdependent in MDA-MB-231 breast cancer cells. Overexpression of MCT4 and/or CD147 increased, and their knockdown decreased, migration, invasion and the degradation of fluorescently labeled gelatin. Overexpression of both proteins led to increases in gelatin degradation and appearance of the matrix metalloproteinase (MMP)-generated collagen-I cleavage product reC1M, and these increases were greater than those observed upon overexpression of each protein alone, suggesting a concerted role in ECM degradation. MCT4 and CD147 colocalized with invadopodia markers at the plasma membrane. They also colocalized with MMP14 and the lysosomal marker LAMP1, as well as partially with the autophagosome marker LC3, in F-actin-decorated intracellular vesicles. We conclude that MCT4 and CD147 reciprocally regulate each other and interdependently support migration and invasiveness of MDA-MB-231 breast cancer cells. Mechanistically, this involves MCT4–CD147-dependent stimulation of ECM degradation and specifically of MMP-mediated collagen-I degradation. We suggest that the MCT4–CD147 complex is co-delivered to invadopodia with MMP14.

## Teaser

**Highlighted Article:** We show that MCT4 and CD147 colocalize with MMP14 at invadopodia and intracellular sites of ECM degradation, and favor breast cancer cell invasion by stimulating ECM degradation.

## INTRODUCTION

Despite major advances in therapy in recent decades, breast cancer remains the leading cause of cancer mortality in women worldwide (Sung et al., 2021). Metastasis to bone, lungs, liver and brain account for ∼90% of breast cancer deaths (Liang et al., 2020; Weigelt et al., 2005), highlighting the major unmet need for improved therapeutic strategies targeting mechanisms of metastasis in this disease. Although the metastatic cascade through which breast cancer cells eventually colonize a distant organ and establish a metastasis involves multiple steps, a fundamental prerequisite is the ability of the cancer cells to remodel and degrade extracellular matrix (ECM), enabling them to invade through surrounding stroma and tissues (Mego et al., 2010; Lu et al., 2011).

The monocarboxylate transporter MCT4 (SLC16A3) is typically strongly expressed in highly glycolytic tissues, where it exports lactate and H^+^ derived from fermentative glycolysis, driven by the combined chemical gradients of these metabolites (Halestrap, 2013; Payen et al., 2020). The plasma membrane localization and, thus, the transport function of MCT4 are dependent on its chaperone CD147 (also known as EMMPRIN, basigin or BSG), a single-pass transmembrane protein with multiple glycosylated states (Grass et al., 2014; Kirk et al., 2000). Conversely, CD147 expression has been shown to be reduced upon knockdown (KD) of MCT4 (Gallagher et al., 2007; Andersen et al., 2018), indicating that the two proteins are interdependent. CD147 has also been assigned a role in binding of carbonic anhydrase IX (CA9) to the CD147–MCT4 complex, facilitating lactate–H^+^ transport (Ames et al., 2020).

Both MCT4 and CD147 are upregulated in many cancers, and their expression correlates with poor prognosis (Payen et al., 2020; Xin et al., 2016). In the case of MCT4, this is often attributed to a role in enabling the high fermentative glycolytic rates common in aggressive cancers, which hence enables tumor growth (Morais-Santos et al., 2015; Le Floch et al., 2011; Andersen et al., 2018). However, more recently, we and others have demonstrated roles for MCT4 in cancer cell migration and invasiveness (Gallagher et al., 2009; Kong et al., 2016; Zhu et al., 2014). Although a role for MCT4 interaction with integrins has been proposed to favor cell migration (Gallagher et al., 2009), the mechanisms through which MCT4 favors invasiveness remain essentially unelucidated.

It has long been appreciated that CD147 facilitates cancer invasiveness in a manner linked to production and activation of several matrix metalloproteinases (MMPs) (Grass et al., 2012; Sun and Hemler, 2001). However, the mechanisms proposed to be involved in this have been contentious, including whether the ability of CD147 to regulate MMPs is independent from its role as an MCT chaperone (Schneiderhan et al., 2009). CD147 can reside in the plasma membrane, as well as in subcellular compartments, and can be released from cells in various forms (Albrechtsen et al., 2019; Grass and Toole, 2015; Egawa et al., 2006; Taylor et al., 2002; Ko et al., 2023). The relation between CD147 glycosylation state, localization and function is incompletely understood, and both high-glycosylated (HG) and low-glycosylated (LG) forms of CD147 have been suggested to have the capacity for activating MMPs, although the HG form might do so more effectively (Huang et al., 2013; Bai et al., 2014). Finally, CD147 interacts with multiple other proteins with roles in cancer cell invasion, including CD44 (Grass et al., 2014), caveolin-1 and several integrins (Berditchevski et al., 1997; Tang and Hemler, 2004). Thus, whether the roles of MCT4 and CD147 in cancer cell motility are dependent on their interaction remains unknown.

The aim of the present study, therefore, is to test the hypothesis that MCT4 and CD147 favor breast cancer invasiveness through interdependent effects on ECM degradation. We show that MCT4 and CD147 expression and localization are highly interdependent, and that MCT4 and CD147 colocalize not only in the plasma membrane but also in characteristic F-actin-decorated vesicles. Overexpression (OE) of MCT4 and/or CD147 increases, and their KD decreases, invasiveness and ECM degradation. We suggest that the interaction of MCT4 and CD147 in both the plasma membrane and F-actin-decorated vesicles is important for the role of these proteins in breast cancer cell invasiveness.

## RESULTS

### Protein expression levels of MCT4 and CD147 are reciprocally interdependent

**Fig. 1.**
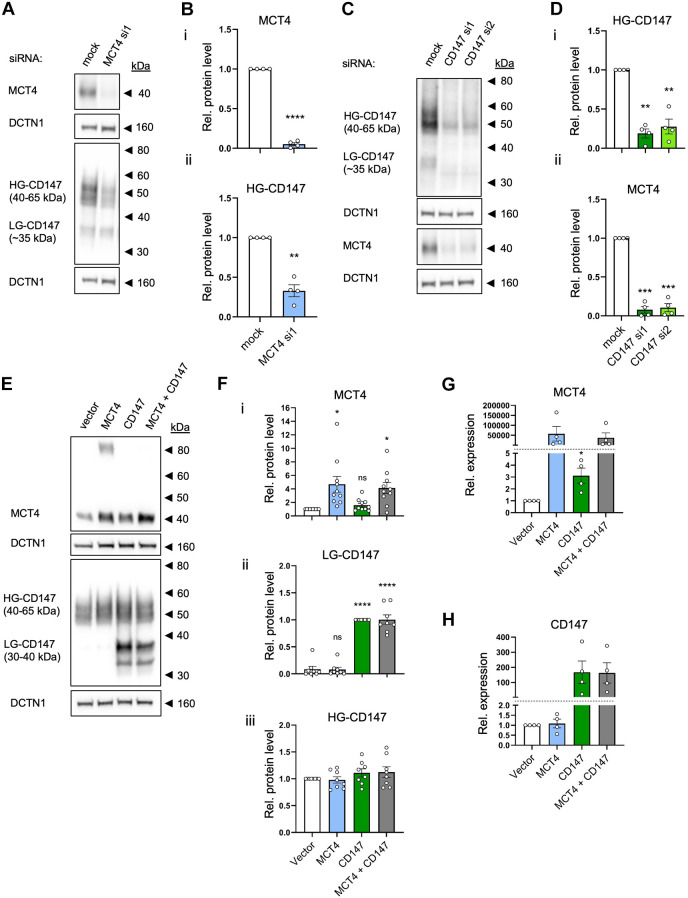
**Protein expression levels of MCT4 and CD147 are reciprocally interdependent.** (A–D) MDA-MB-231 cells were transfected with siRNA (si) targeting MCT4 (A,B) or CD147 (C,D), or with scrambled siRNA as a control (mock). At 48 h post-transfection, cells were lysed and subjected to western blotting. Representative western blots (A,C) and corresponding quantification of MCT4 and HG CD147 protein levels (B,D) are shown. DCTN1 was used as loading control. MCT4 and HG CD147 band intensities were normalized to their respective loading controls and control (mock). *n*=4. (E–H) MDA-MB-231 cells were transfected with MCT4, CD147, or MCT4 and CD147 (MCT4+CD147) OE plasmids, or with empty pcDNA3.1 (+) vector as negative control (vector). At 24 h post-transfection, cells were lysed and subjected to western blot (E,F) or qPCR analysis (G,H). (E,F) Representative western blots (E) and corresponding quantification (F) of MCT4 and CD147 protein levels. DCTN1 was used as loading control. MCT4 and CD147 band intensities were normalized to loading controls and control (vector). *n*=10 (MCT4), *n*=8 (CD147). (G,H) Relative mRNA levels (determined by qPCR) of MCT4 and CD147. Data was normalized to housekeeping genes β-actin and *TBP*, and to the vector control. *n*=4. Quantitative data are presented as mean±s.e.m. *P<0.05; **P<0.01; ***P<0.001; ****P<0.0001; ns, not significant (two-tailed paired Student's *t*-test in B and G, one-way ANOVA with Dunnett's post-hoc test in D,F and H). Rel., relative.

The first step in understanding the possible relationship between MCT4 and CD147 in regulation of breast cancer cell motility was to determine their interrelationship at the protein level. Western blotting showed that MCT4 protein expression was essentially ablated 48 h after transfection of MDA-MB-231 human breast cancer cells with siRNA targeting MCT4 (Fig. 1A,Bi). Notably, this MCT4 KD was associated with a strong reduction in expression of the HG form of CD147 (Fig. 1A,Bii). Conversely, KD of CD147 using two different siRNAs not only strongly attenuated CD147 expression but also essentially ablated MCT4 expression (Fig. 1C,D). A different pattern emerged when MCT4 or CD147 were instead transiently overexpressed. OE of MCT4 (using rat MCT4, which is 89% similar to human MCT4 at the protein level), but not of CD147, significantly increased the MCT4 protein level (Fig. 1E,Fi). Similarly, OE of CD147 increased the protein level of CD147, and this increase was only significant for the LG form (Fig. 1E,Fii,Fiii). When MCT4 and CD147 were co-expressed using the same total amount of cDNA (i.e. 50% of each), the expression levels of both proteins were found to be the same as those observed following individual OE; however, a misfolded MCT4 dimer band (Wilson et al., 2009) disappeared upon co-expression (Fig. 1E,F). No changes in overall morphology of the cells were detected upon MCT4 and/or CD147 OE or KD (Fig. S1).

To further interrogate the apparent tendency for an increase in MCT4 level upon CD147 expression (Fig. 1Fi), we determined the mRNA levels of MCT4 (*SLC16A3*) and CD147 (*BSG*) by qPCR analysis (Fig. 1G,H). The pattern of changes was similar to that seen at the protein level, but interestingly, we observed a minor but significant increase in MCT4 mRNA level upon CD147 OE (Fig. 1G).

The apparent lack of interdependence of the mean MCT4 and CD147 protein levels in the cell population after OE (contrasting with the marked effect of KD) might reflect that not all cells were transfected (average transfection efficacy of 8.6% for MCT4 and 14.2% for CD147, estimated from immunofluorescence analysis; *n*=4). We reasoned that the effect of OE would be better resolved in a cell line with lower basal expression of both proteins than MDA-MB-231 cells, and we therefore repeated the experiments in MCF10A non-cancer mammary epithelial cells, in which CD147 OE has previously been shown to lead to invasiveness and formation of filopodia-like structures (Grass et al., 2012) (Fig. S2). In these cells, endogenous levels of MCT4 in particular were much lower than in MDA-MB-231 cells, and although OE of CD147 alone did not increase MCT4 expression, co-expression of MCT4 with CD147 resulted in much higher levels of MCT4 than were observed upon OE of MCT4 alone, and strongly reduced levels of the misfolded dimer band (Fig. S2A). In these cells, co-expression furthermore appeared to increase levels of the HG form of CD147. This pattern was confirmed by quantification of mean levels of MCT4 (Fig. S2B) and of the LG and HG forms of CD147 (Fig. S2C,D). Finally, consistent with previous reports (Gallagher et al., 2007; Kirk et al., 2000; Deora et al., 2005), MCT4 and CD147 reciprocally co-immunoprecipitated in MDA-MB-231 cells, confirming their physical interaction (Fig. S2E–G). Taken together, these results show that protein levels of MCT4 and CD147, and most likely also the correct folding and maturation of the two proteins, are reciprocally interdependent.

### Overexpression increases MCT4 and CD147 plasma membrane localization

**Fig. 2.**
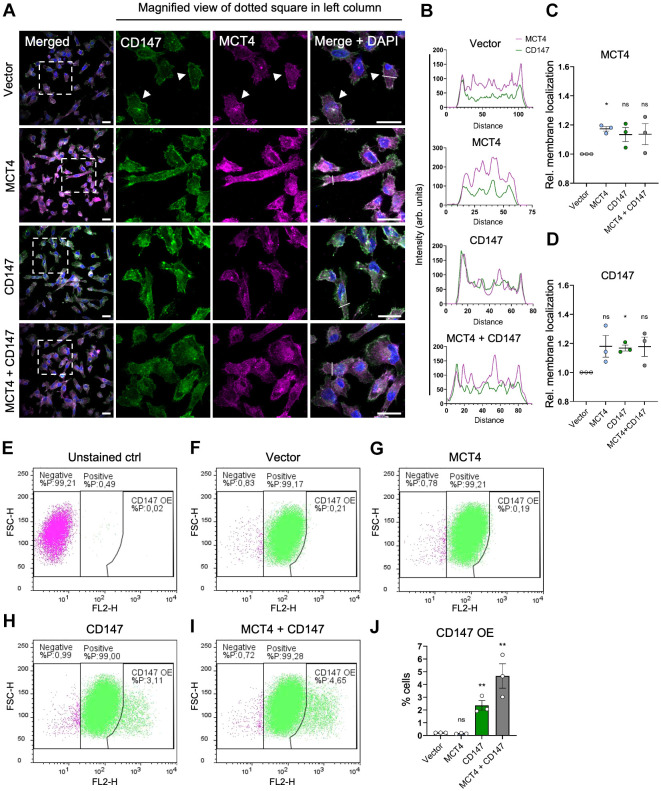
**Overexpression increases MCT4 and CD147 plasma membrane localization.** (A) Representative overview images (left) and magnified views (right) of MDA-MB-231 cells transfected with empty vector, or with MCT4, CD147, or MCT4 and CD147 (MCT4+CD147) OE plasmids. Cells were grown for 24 h, fixed and subjected to IFM analysis with primary antibodies targeting MCT4 (magenta) and CD147 (green). Nuclei were stained with DAPI (blue). Dashed box indicates region shown in magnified views. White arrowheads show colocalization of MCT4 and CD147 in the plasma membrane, and white lines represent the regions analyzed by line scan analysis in B. *n*=3. Scale bars: 20 µm. (B) Representative line scan analysis plots showing intensities (MCT4, magenta; CD147, green) along a selected line (white lines in A) for the indicated OE and vector control cells (arb. units, arbitrary units; distances shown in pixels). (C,D) Relative membrane localization of MCT4 (C) and CD147 (D), determined by line scan analysis. Color intensities were normalized to cell count and to their respective control (vector). Mean±s.e.m. of *n*=3 independent biological experiments, with 61–109 line scans performed for each experiment and condition. (E–I) Representative flow cytometric dot plots [FSC versus PE-conjugated anti-human CD147 signal (FL2-H)]. Gates for CD147-negative cells, CD147-positive cells and CD147 OE cells are marked in the plots and were set based on an unstained empty vector control (E) and an antibody-stained empty vector control (F). The percentage of cells in each population for the controls and for cells transfected with MCT4 (G), CD147 (H), and MCT4 and CD147 (I) OE plasmids are shown in the respective plots. Dot plots are representative of *n*=3. (J) Quantification of CD147 OE populations as a percentage of total cells, as determined by the flow cytometry assay. Statistical analysis was performed on log-transformed data. Mean±s.e.m. of *n*=3. *P<0.05; **P<0.01; ns, not significant (one-way ANOVA with Dunnett's post-hoc test). Rel., relative.

We next asked whether OE of MCT4 and/or CD147 led to upregulation of functional, plasma membrane-localized proteins. We overexpressed the two proteins alone or together, as before, followed by immunofluorescence microscopy (IFM) analysis of total levels of both proteins (endogenous and overexpressed) (Fig. 2A). MCT4 and CD147 were visible both in intracellular structures and in the plasma membrane (Fig. 2A, arrowheads), where they partially colocalized both endogenously and after OE. To quantify membrane localization, we first performed line scan analysis of the IFM images (experimental conditions were masked during the analysis; for details, please refer to the Materials and Methods). Determined in this manner, OE of MCT4 and/or CD147 was found to increase, or tend to increase, plasma membrane localization of both proteins (Fig. 2C,D). As seen in the magnified views of IFM images (Fig. 2A) and illustrated by representative line scan profiles (Fig. 2B), OE was also associated with increased intracellular levels of each protein. This was particularly prominent for OE of MCT4 alone, which is consistent with the misfolded MCT4 dimer seen by western blotting (Fig. 1), but was also detectable for OE of CD147 alone and for the co-expressed proteins, consistent with the high level of the LG form of CD147 observed in the assays shown in Fig. 1.

Flow cytometric analysis of the plasma membrane level of CD147 using an independent, directly phycoerythrin (PE)-conjugated antibody confirmed increased membrane localization of CD147 upon OE and suggested that co-expression with MCT4 further increased CD147 membrane localization (Fig. 2E–J).

These results show that MCT4 and CD147 localize both intracellularly and to the plasma membrane, partially colocalizing in both locations, and that plasma membrane localization of both proteins is increased upon OE.

### Invasion of MDA-MB-231 cells is dependent on MCT4 and CD147

Both MCT4 (Kong et al., 2016; Zhu et al., 2014) and CD147 (Grass et al., 2012; Sun and Hemler, 2001) have been implicated in cancer cell invasion; however, their interdependence in this regard has not been addressed, and the underlying mechanisms are unclear. To gain insight into this, we knocked down and overexpressed both proteins, alone and in combination, and studied the effects on MDA-MB-231 cell motility.

**Fig. 3.**
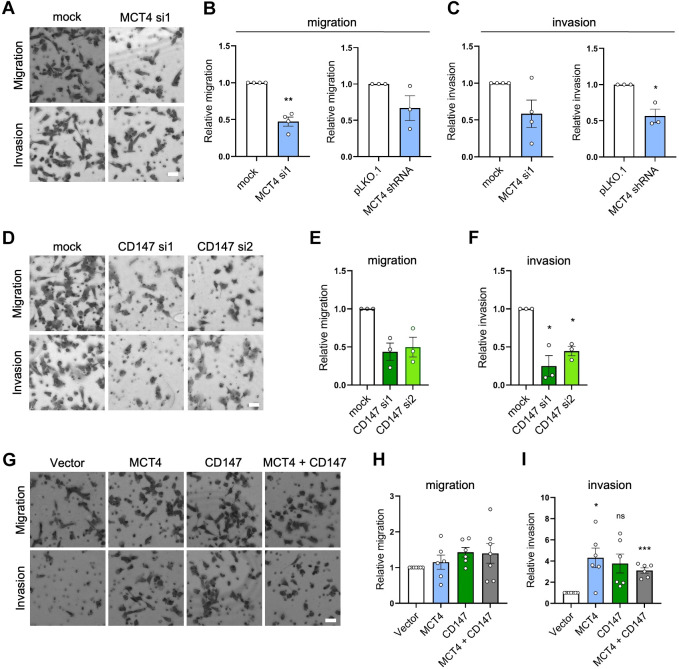
**MCT4 and CD147 are both important for the invasiveness of MDA-MB-231 cells.** (A–C) MDA-MB-231 cells were transfected with siRNA (si) targeting MCT4 or with scrambled siRNA as a control (mock). Alternatively, MDA-MB-231 cells with stable KD of MCT4 were employed (MCT4 shRNA) alongside a control MDA-MB-231 cell line (pLKO.1). Cells were seeded in migration or invasion Boyden chamber inserts with 10% FBS as chemoattractant in the lower chamber, and migration or invasion, as indicated, was determined 24 h after seeding of the cells in the upper chamber. Cells were fixed and stained with Giemsa solution, and relative cell migration or invasion was determined by counting the cells that had moved through the inserts. (A) Representative images of control and MCT4 siRNA-treated cells in the two assays. (B) Relative cell migration as determined by the number of migrated cells relative to that in the control condition. (C) Relative cell invasion as determined by the number of cells invaded relative to that in the control condition. *n*=4 for siRNA KD experiment, *n*=3 for shRNA KD experiment. (D–F) MDA-MB-231 cells were transfected with siRNA targeting CD147 or with scrambled siRNA as a control, and were then used in migration and invasion assays as described for A–C. (D) Representative images of control and CD147 siRNA-treated cells in the two assays. (E) Relative cell migration as determined by the number of migrated cells relative to that in the control condition. (F) Relative cell invasion as determined by the number of cells invaded relative to that in the control condition. *n*=3. (G–I) MDA-MB-231 cells were transfected with MCT4, CD147, or MCT4 and CD147 (MCT4+CD147) OE plasmids, or with empty vector as a control (vector), and were then used in migration and invasion assays as described for A–C. (G) Representative images of control and OE cell lines in the two assays. (H) Relative cell migration as determined by the number of migrated cells relative to that in the control condition. (I) Relative cell invasion as determined by the number of cells invaded relative to that in the control condition. *n*=6. Quantitative data are presented as mean±s.e.m. *P<0.05; **P<0.01; ***P<0.001; ns, not significant (two-tailed, paired Student's *t*-test in B and C; one-way ANOVA with Dunnett's post-hoc test in E,F,H and I). Scale bars: 40 µm.

Transient siRNA-mediated KD of MCT4 (which reduced MCT4 protein level by ∼90% and HG CD147 protein level by ∼70%; see Fig. 1B) reduced MDA-MB-231 cell migration and invasion by ∼50% at 24 h after seeding in Boyden chamber assays (Fig. 3A–C). Confirming this result, stable shRNA-mediated KD of MCT4, which reduced MCT4 and HG CD147 protein levels by ∼90% and ∼70%, respectively (Fig. S3A–C), similarly reduced cell migration and invasion (Fig. 3B,C). This effect was not due to an impact of MCT4 KD on cell proliferation, which, although decreased after transient KD of MCT4, was unaltered after stable MCT4 KD (Fig. S3D). When we instead knocked down CD147 (reducing HG CD147 and MCT4 protein levels by ∼80% and ∼90%, respectively; Fig. 1D), a similar reduction in migration and invasion was observed, using two different siRNAs (Fig. 3D–F) and without effect on proliferation (Fig. S3E). Finally, when MCT4, CD147 or both proteins were overexpressed in MDA-MB-231 cells, and migration and invasion were determined 24 h after seeding, invasion was found to be increased approximately fourfold (although the increase was not significant in cells transfected with CD147 alone due to substantial variation), whereas migration was, interestingly, not significantly increased (Fig. 3G–I). This, again, was not due to effects on proliferation, which was unaffected in the OE conditions (Fig. S3F). These results show that MCT4 and CD147 are necessary for MDA-MB-231 cell migration and invasion, and that their OE further increases invasion.

### MCT4 and CD147 are important for ECM degradation

**Fig. 4.**
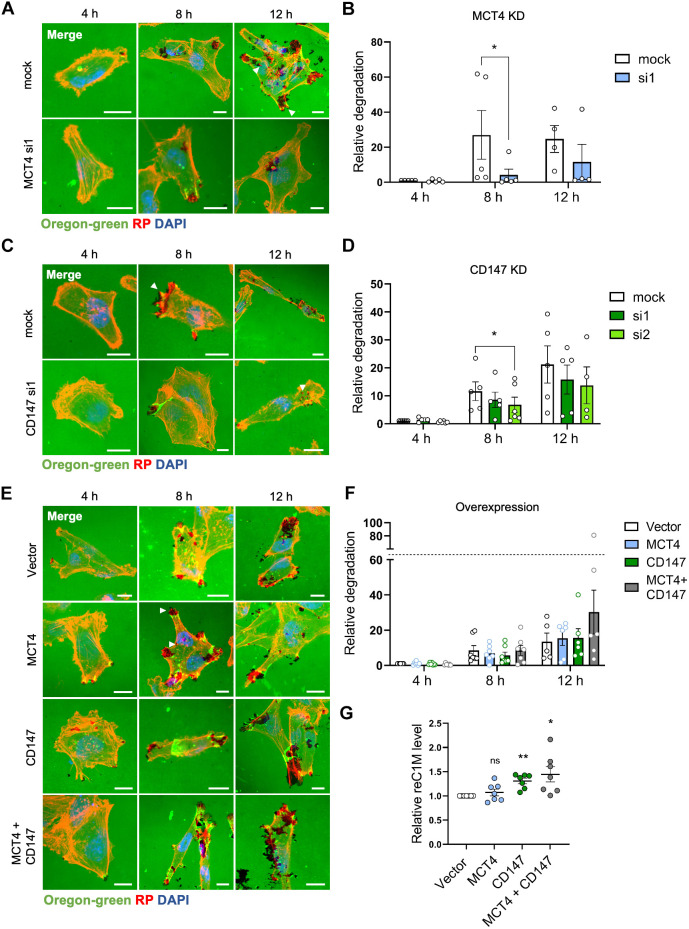
**MCT4 and CD147 are important for ECM degradation.** (A–F) MDA-MB-231 cells were transfected with siRNA (si) targeting MCT4 or scrambled siRNA as a control (mock) (A,B); with siRNA targeting CD147 or scrambled siRNA as a control (C,D); or with either MCT4, CD147, or MCT4 and CD147 (MCT4+CD147) OE plasmids, or with empty vector as a control (E,F). Transfected cells were seeded on coverslips coated with Oregon Green 488-conjugated gelatin and grown for 4, 8 or 12 h, as indicated. Cells were subsequently fixed and stained for F-actin (using Rhodamine–phalloidin, RP) and nuclei (DAPI). Fig. S4 shows the corresponding full overview images alongside the cropped images shown in this figure, as well as the images for CD147 siRNA-2 (si2). (A,C,E) Representative images of cells growing on Oregon Green 488-conjugated gelatin for the indicated times, presented as merged images of Oregon Green 488 (green), F-actin (RP; red) and DAPI (blue). White arrowheads indicate overlap of gelatin degradation with F-actin staining at cell protrusions and intracellular structures. Scale bars: 10 µm. (B,D,F) Quantification of gelatin degradation by the indicated MDA-MB-231 cells after 4, 8 and 12 h. Relative degradation is the degraded area fraction normalized to the cell count. The data points represent the mean relative degradation of 6–13 images per condition. *n*=4 or 5 for MCT4 KD (B), *n*=4–6 for CD147 KD (D), *n*=5–7 for OE (F). (G) Relative reC1M (MMP-generated fragment of collagen-I) concentration measured by ELISA in samples collected from the culture medium of MDA-MB-231 cells (transfected with empty vector or with MCT4, CD147, or MCT4 and CD147 OE plasmids, as indicated) 48 h after seeding on collagen-I. reC1M concentrations are normalized to the vector control within each independent experiment. *n*=7. Quantitative data are presented as mean±s.e.m. *P<0.05; **P<0.01; ns, not significant (two-tailed paired Student's *t*-test in B, one-way ANOVA with Dunnett's post-hoc test for each time point in D and F, one-way ANOVA with Dunnett's post-hoc test in G; analyses were performed on log-transformed data).

The ability of cancer cells to degrade the surrounding ECM is an essential element of their invasiveness. ECM degradation has previously been shown to be regulated by other net acid-extruding transporters, at least in part because of the acidic pH optimum of MMPs (Busco et al., 2010; Pedraz-Cuesta et al., 2016). Given the role of MCT4 in coupled extrusion of lactate and H^+^, we therefore reasoned that the transporter could support invasiveness by facilitating ECM degradation. To test this hypothesis, we established a matrix degradation assay based on the ability of the cells to degrade Oregon Green 488-conjugated gelatin, a collagen derivative with structural and functional properties similar to those of collagens (Bello et al., 2020). MDA-MB-231 cells were seeded on the fluorescently labeled gelatin, and live imaging experiments established that cells actively degrade the matrix under them, as they move on the coverslip (see Movie 1 for fluorescence imaging and Movie 2 for corresponding bright-field imaging). Subsequently, cells were transfected with the relevant siRNAs or OE plasmids; fixed after 4, 8 or 12 h; and stained with Rhodamine–phalloidin and DAPI to visualize F-actin and nuclei, respectively (zoomed-in images are shown in Fig. 4; full overview images are shown in Fig. S4). Mock-transfected cells efficiently degraded the gelatin – evident as the gradual disappearance of Oregon Green 488 fluorescence over time – particularly under actin-rich cellular structures, including both protrusions and apparently intracellular regions (Fig. 4A, white arrowheads). In contrast, after MCT4 KD, cells appeared to degrade substantially less gelatin. Quantification of the degraded areas and normalization to cell count showed that gelatin degradation at 8 h was reduced by more than 80% by MCT4 KD (Fig. 4B). CD147 KD similarly decreased gelatin degradation under actin-rich structures (Fig. 4C, white arrowheads) and reduced gelatin degradation at 8 h, although only by ∼50% (Fig. 4C,D). MCT4 or CD147 KD also tended to reduce degradation at 12 h after seeding, although extensive experimental variability at this time precluded statistical significance (Fig. 4A–D). In contrast, OE of MCT4 or CD147 alone, or of both proteins together, did not alter gelatin degradation to a statistically significant degree, although a tendency for an increase in degradation was seen at 12 h upon combined OE (Fig. 4E,F).

Taken together, these experiments strongly indicated that collagen degradation is dependent on MCT4 and CD147. To confirm this and to gain insight into the nature of the degrading protease, we next overexpressed MCT4, CD147 or both proteins in MDA-MB-231 cells as above, seeded the cells on collagen-I, and determined the appearance of reC1M – a collagen-I neoepitope generated specifically through collagen-I degradation by MMP2, MMP9 or MMP13 (Leeming et al., 2011) – in the culture medium by enzyme-linked immunosorbent assay (ELISA) 48 h after seeding (Fig. 4G). Notably, whereas the increase in reC1M levels after MCT4 OE alone did not reach statistical significance, reC1M levels were significantly increased upon OE of CD147 or both proteins together. These results show that MCT4 and CD147 are important for the degradation of the ECM by MDA-MB-231 cells, and that this involves MMP-dependent degradation of collagen-I.

### MCT4 and CD147 colocalize to ECM-degrading invadopodia and MMP14-containing intracellular structures

Having shown that MCT4 and CD147 are important for matrix degradation by MDA-MB-231 cells, we wished to determine whether the two proteins colocalized in actively degrading invadopodia. To increase formation of invadopodia, we overexpressed constitutively active Src (Src Y527F) (Seals et al., 2005; Artym et al., 2006). Co-staining for Src, F-actin and cortactin phosphorylated at Y421 (pY421-cortactin), which localizes to invadopodia and is important for their function (Jeannot and Besson, 2020), confirmed the strong localization of pY421-cortactin to invadopodia (Fig. S5A). We could thus use pY421-cortactin as an invadopodia marker, and subsequent co-staining of pY421-cortactin with MCT4 and CD147 showed that both proteins prominently, albeit only partially, colocalize to invadopodia (Fig. S5B, white arrows).

**Fig. 5.**
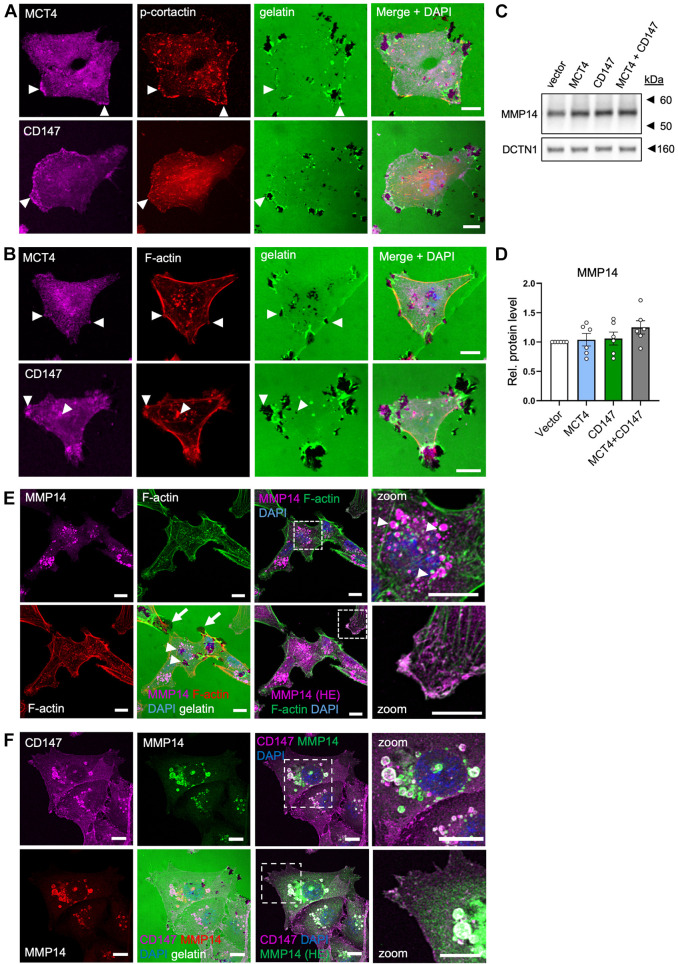
**MCT4 and CD147 colocalize to ECM-degrading invadopodia and MMP14-containing intracellular structures.** (A,B) Wild-type MDA-MB-231 cells were seeded on coverslips coated with Oregon Green 488-conjugated gelatin, fixed after 8 h of growth and subsequently subjected to IFM analysis. Nuclei were stained with DAPI (blue). Representative images of MDA-MB-231 cells on gelatin (green) showing the cellular localization of MCT4 (magenta) or CD147 (magenta), and either pY421-cortactin (A, red) or F-actin (B, red). White arrowheads indicate overlap of gelatin degradation with MCT4 or CD147 and with pY421-cortactin (A) or F-actin (B). *n*=3. (C,D) Representative western blot (C) and corresponding quantification (D) of MMP14 in MDA-MB-231 cells transfected with MCT4, CD147, or MCT4 and CD147 (MCT4+CD147) OE plasmids, or with empty vector as a control (vector). DCTN1 was used as loading control. MMP14 band intensities were normalized to the loading control and vector control. Data in D are presented as mean±s.e.m. of *n*=6. Rel., relative. (E) Wild-type MDA-MB-231 cells were seeded on coverslips coated with Oregon Green 488-conjugated gelatin, fixed after 8 h of growth and subsequently subjected to IFM analysis. Nuclei were stained with DAPI (blue). Representative images of MDA-MB-231 cells on gelatin (green, lower panel) showing the cellular localization of MMP14 (magenta) and F-actin (green or red). MMP14 is shown at a low (upper panel) and high (HE, lower panel) exposure. Dashed boxes indicate the regions shown in zoom images. Arrowheads in the top zoom panel indicate MMP14-containing, F-actin-decorated vesicles. Arrowheads in the lower panel indicate matrix degradation overlapping with these vesicles. Arrows indicate matrix degradation at cell protrusions. *n*=3. (F) Wild-type MDA-MB-231 cells were seeded on coverslips coated with Oregon Green 488-conjugated gelatin, fixed after 8 h of growth and subsequently subjected to IFM analysis. Nuclei were stained with DAPI (blue). Representative images of MDA-MB-231 cells on gelatin (green, lower panel) showing the cellular localization of CD147 (magenta) and MMP14 (red or green). MMP14 (green) is shown at a low (upper panel) and high (HE, lower panel) exposure. Dashed boxes indicate the regions shown in zoom images. *n*=3. Scale bars: 10 µm.

To determine whether endogenous MCT4 and CD147 localized to actively degrading invadopodia in non-Src-transformed cells, cells were cultured on Oregon Green 488-conjugated gelatin for 8 h and stained for MCT4 or CD147, and either pY421-cortactin (Fig. 5A) or F-actin (Fig. 5B). This revealed clear localization of both proteins to structures rich in pY421-cortactin and F-actin, colocalizing with marked matrix degradation (Fig. 5A,B, white arrowheads). Whereas some of these structures were reminiscent of lamellipodia or other focal adhesion-rich protrusions, others were intracellular – either vesicular or found at the interface between cells and the matrix (Fig. 5B, lower row, arrowhead). Co-immunofluorescence analysis with F-actin, LAMP1 (a marker of lysosomes), protein disulfide-isomerase [PDI, a marker of the endoplasmic reticulum], golgin-97 (also known as GOLGA1; a Golgi marker) and TOM20 (also known as TOMM20; a mitochondrial marker) indicated that CD147 and MCT4 were mainly associated with F-actin-containing structures and with lysosomes, arguing against major accumulation in the endoplasmic reticulum or Golgi (Fig. S6).

MMP14 is overexpressed in many cancers and is a major upstream activator of other MMPs. These include MMP2 (Itoh et al., 2001) and MMP13 (Knäuper et al., 2002), two of the MMPs generating the reC1M neoantigen (Leeming et al., 2011). Total MMP14 expression was not significantly altered by OE of MCT4, CD147 or both proteins (Fig. 5C,D). Growth of the MDA-MB-231 cells on Oregon Green 488-conjugated gelatin followed by staining for MMP14 and F-actin revealed that, although also expressed at the plasma membrane (Fig. 5E, high exposure), MMP14 was most strongly localized to intracellular vesicles that each appeared characteristically coupled to F-actin ‘dots’ (Fig. 5E, top panel, white arrowheads). Co-staining of MMP14 with CD147 on Oregon Green 488-conjugated gelatin revealed clear colocalization between MMP14 and CD147 in these intracellular vesicles, and higher exposure also revealed colocalization at the plasma membrane (Fig. 5F). Matrix degradation was occasionally detectable associated with these structures (Fig. 5E, lower panel, arrowheads) but was more frequently seen at the cell protrusions (Fig. 5E, lower panel, arrows).

These results show that CD147 and MMP14 colocalize to matrix-degrading protrusions as well as to large intracellular vesicles decorated by F-actin ‘dots’. By comparison with the colocalization of CD147 and MCT4 at these structures (Fig. 2A, Fig. 5A; Fig. S6), it can be inferred that MCT4 is also present at the structures containing CD147 and MMP14.

### MCT4 and CD147 colocalize with MMP14 in LC3-positive vesicles

**Fig. 6.**
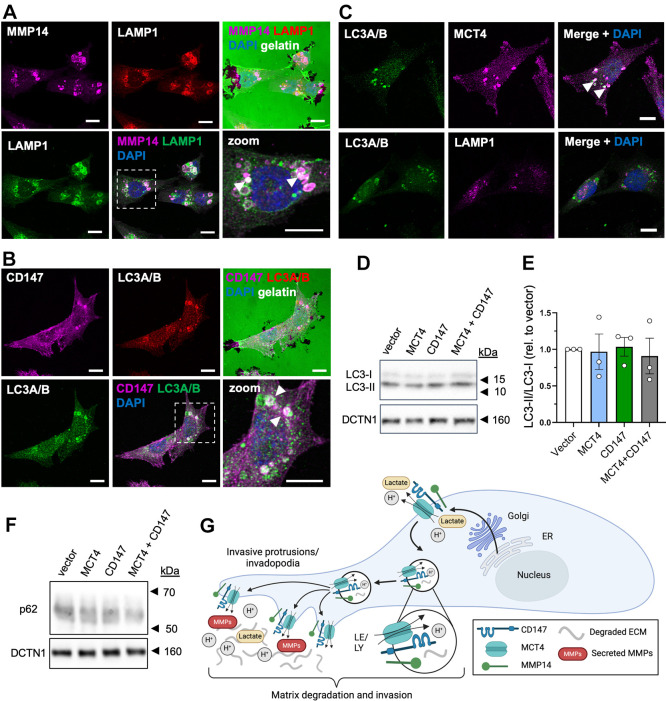
**MCT4 and CD147 colocalize with MMP14 in LC3-positive vesicles.** (A–C) Wild-type MDA-MB-231 cells were seeded on coverslips coated with Oregon Green 488-conjugated gelatin, fixed after 8 h of growth and subsequently subjected to IFM analysis. Nuclei were stained with DAPI (blue). (A) Representative images of MDA-MB-231 cells on gelatin (green, upper panel) showing the cellular localization of MMP14 (magenta) and LAMP1 (red in upper panel, green in lower panel). Dashed box indicates region shown in the zoom image. Arrowheads in lower right image indicate vesicles co-stained for MMP14 and LAMP1. *n*=3. (B) Representative images of MDA-MB-231 cells on gelatin (green, upper panel) showing the cellular localization of CD147 (magenta) and LC3A/B (red in upper panel, green in lower panel). Dashed box indicates region shown in the zoom image. Arrowheads in lower right image indicate vesicles co-stained for LC3A/B and CD147. *n*=3. (C) Representative images showing the localization of LC3A/B (green) and MCT4 (magenta, top panel), and of LC3A/B (green) and LAMP1 (magenta, lower panel). Arrowheads in upper right image indicate vesicles co-stained for LC3A/B and MCT4. *n*=1. Scale bars: 10 µm. (D,E) Representative western blot (D) and corresponding quantification (E) of LC3-II: LC3-I ratio 48 h after transfection with MCT4, CD147, or MCT4 and CD147 (MCT4+CD147) OE plasmids, or with empty pcDNA3.1 (+) vector as negative control (vector). DCTN1 was used as loading control. Quantitative data are presented as mean±s.e.m. of *n*=3. Rel., relative. (F) Representative western blot of p62 protein level 48 h after transfection with MCT4, CD147, or MCT4 and CD147 OE plasmids, or with empty pcDNA3.1 (+) vector as negative control. DCTN1 was used as loading control. *n*=3. (G) Schematic illustrating our working hypothesis. MCT4–CD147 co-structures are delivered to the plasma membrane along with MMP14, and from there, the three proteins are retrieved together by endocytosis and delivered to invadopodia in large vesicles of late endosomal and lysosomal origin. Whether the role of MCT4–CD147 in ECM degradation reflects an involvement in formation or recycling of the large vesicles and/or lactate–H^+^ efflux in invadopodia remains to be determined. ER, endoplasmic reticulum; LE, late endosome; LY, lysosome.

MMP14 in MDA-MB-231 cells is mainly localized to late endosomes and lysosomes, from where it is trafficked to invadopodia (Monteiro et al., 2013; Poincloux et al., 2009). The striking F-actin ‘dots’ on the MMP14- and CD147-positive vesicles were reminiscent of the F-actin comet-bearing vesicles formed during phagocytosis, macropinocytosis (Mylvaganam et al., 2021) and lysosome–autophagosome fusion (Nakamura and Yoshimori, 2017; Kast and Dominguez, 2017). Furthermore, MMP14 is known to play a critical role in collagen-I phagocytosis (Lee et al., 2006). Co-immunofluorescence analysis of MMP14 and LAMP1 showed substantial staining overlap (Fig. 6A, arrowheads in bottom-right image), confirming the localization of MMP14 to late endosomes and/or lysosomes. Interestingly, some gelatin degradation overlapped with these organelles containing both MMP14 and LAMP1 (Fig. 6A, arrowhead in top-right image). A fraction of the vesicles (Fig. 6B, arrowheads; here stained by CD147) were also positive for microtubule-associated protein 1 light-chain 3 proteins (MAP1LC3A and MAP1LC3B, herein referred to collectively as LC3 or LC3A/B), suggesting that they could be autophagosomes, although other interpretations are possible (Runwal et al., 2019). Furthermore, MCT4 colocalized with LC3 in intracellular vesicles (Fig. 6C, arrowheads), whereas colocalization of LC3 with LAMP1 appeared to be minimal (Fig. 6C, bottom row).

An increased ratio of the lipidated, autophagosome-associated form of LC3 (LC3-II) to the unlipidated form (LC3-I) and a decrease in protein level of the p62 (SQSTM1) autophagy receptor (which is degraded during autophagy of its recruited targets) is indicative of increased autophagy. If MCT4 and CD147 played a role in the phagocytosis and intracellular degradation of collagen-I in intracellular vesicles, and this represented a sizable fraction of total cellular autophagy, then OE of MCT4 and CD147 could increase total cellular autophagic flux. However, neither the LC3-II:LC3-I ratio nor the protein level of the p62 autophagy receptor were altered by OE of MCT4, CD147 or both proteins together (Fig. 6D–F). These results show that MCT4, CD147 and MMP14 colocalize with LC3 in intracellular vesicles, but that their OE does not detectably impact total cellular autophagy.

## DISCUSSION

Increased expression of MCT4 or CD147 correlates with poor prognosis in many cancers (Payen et al., 2020; Xin et al., 2016), and previous work from us and others has established roles for both MCT4 (Kong et al., 2016; Zhu et al., 2014) and CD147 (Grass et al., 2012; Sun and Hemler, 2001) in cancer cell migration and invasion. However, the mechanisms for this remain essentially unelucidated, and the interdependency of the two proteins for proper expression and localization (Gallagher et al., 2007; Andersen et al., 2018; Morais-Santos et al., 2015) makes it difficult to interpret such findings unless both proteins were accounted for in study designs. Here, we tested the hypothesis that MCT4 and CD147 favor breast cancer cell invasiveness through interdependent effects on ECM degradation. A working hypothesis based on our results is shown in Fig. 6G.

MDA-MB-231 cells lack MCT1 (SLC16A1) due to hypermethylation of its promoter region (Asada et al., 2003), and lactate transport in these cells is therefore mediated by MCT4 (Contreras-Baeza et al., 2019). Consistent with previous reports (Gallagher et al., 2007; Andersen et al., 2018; Morais-Santos et al., 2015), we found that KD of either MCT4 or CD147 nearly ablated the protein expression of the other protein. The chaperone effect was particularly evident for MCT4, where a presumably misfolded (Wilson et al., 2009) dimer form was predominant when the transporter was overexpressed alone, and this dimer essentially disappeared upon co-expression with CD147. Interestingly and unexpectedly, CD147 OE also tripled the mRNA level of MCT4 (but not vice versa). This has, to our knowledge, not previously been reported and indicates that the interrelationship between the two proteins is more complex than previously anticipated. Although not studied here, the simplest interpretation is that the increased protein expression of CD147 might allow increased MCT4 translation, in turn reducing mRNA degradation. Although more difficult to quantify, IFM analysis using line scans and flow cytometry analysis of cell surface CD147 expression also indicated that increased plasma membrane expression of one of the proteins generally increased the plasma membrane expression of the other. Finally, we showed that MCT4 and CD147 co-immunoprecipitate, confirming previous reports of their physical interaction (Gallagher et al., 2007; Kirk et al., 2000; Deora et al., 2005).

Confirming and extending earlier reports by us and others (Gallagher et al., 2009; Kong et al., 2016; Zhu et al., 2014; Andersen et al., 2018), we next showed that MDA-MB-231 cell migration and invasion were attenuated by KD of either MCT4 or CD147, and conversely, were increased by their OE. These effects at least in part involve the transport activity of MCT4, as inhibition of monocarboxylate transporters (Kong et al., 2016) or an MCT4 mutation abolishing transport (Li et al., 2021) attenuate cancer cell migration and invasion. A role for MCT4 interaction with integrins has been proposed to contribute to the role of the transporter in cell migration, based on co-immunoprecipitation and colocalization to the leading edge (Gallagher et al., 2009). However, the mechanisms through which MCT4 favors invasiveness are unknown. MCT4-mediated lactate–H^+^ co-extrusion could, similar to what has been demonstrated for other net acid-extruding transporters, generate a local acidic environment favoring ECM degradation by enhancing protease activity (Stock et al., 2008). Alternatively, or concomitantly with this, the lactate extruded by MCT4 could activate the lactate receptor HCAR1, further enhancing invasiveness (Ishihara et al., 2022).

We therefore hypothesized that a key role for the MCT4–CD147 co-structure in cancer cell invasiveness could be to stimulate ECM degradation. Consistent with our hypothesis, we first showed that degradation of fluorescently labeled gelatin was decreased by KD of MCT4 or CD147 and tended to be increased upon their joint OE. MCT4 and CD147 colocalized strongly to protrusive plasma membrane regions that further co-expressed pY421-cortactin, Src, and F-actin. These were also the regions with the most prominent matrix degradation, identifying them as invasive structures, likely invadopodia. Seeding cells on collagen-I, we next demonstrated that OE of CD147, or even more so of both MCT4 and CD147, increased the appearance of the reC1M collagen-I neoepitope, which is the cleavage product resulting from collagen-I degradation by MMP-2, MMP-9 or MMP-13 (Leeming et al., 2011). In support, clinically, reC1M measured in serum has been found to be associated with poor survival outcomes in two metastatic breast cancer cohorts (Lipton et al., 2018). MMP2 (Itoh et al., 2001) and MMP13 (Knäuper et al., 2002) are activated by MMP14, a key player in cancer cell invasion that is itself involved in direct degradation of ECM (Poincloux et al., 2009). MMP14 expression was, however, not altered by MCT4 and/or CD147 OE, and was in fact mainly localized to large, intracellular vesicle-like structures, although MMP14 was also found in the peripheral invasive structures mentioned above. ECM degradation in cancer cells is not only extracellular, and early studies reported its occurrence in unusually large, very acidic vesicles (LAVs) (Montcourrier et al., 1994, 1990). LAVs have been reported to be phagosomal structures harboring cathepsin D and multivesicular bodies, and as having extremely low pH (∼pH 4, compared to ∼pH 5 in lysosomes; Montcourrier et al., 1994, 1990).

We therefore focused our attention on the intracellular structures, finding that CD147, MCT4 and MMP14 strongly colocalized in them. These structures were very large intracellular vesicles, reminiscent of LAVs, with a characteristic F-actin decoration that reminded us of the actin comet tails on autophagosomes (Kast et al., 2015; Kast and Dominguez, 2017). Consistent with the latter, MCT4, CD147 and MMP14 colocalized with the lysosomal marker LAMP1 and partially with the autophagosomal marker LC3 in intracellular vesicles, suggesting that a fraction of these vesicles might be destined for autophagic degradation. However, the only partial colocalization with LC3, and the fact that MCT4 and/or CD147 OE did not appear to increase total cellular autophagy, seems to argue against a major role of autophagy in MCT4–CD147-facilitated ECM degradation.

Interestingly, MMP14 can be released to the ECM via fusion of late multivesicular exosomes (MVEs) with the plasma membrane at sites of invadopodia (Hoshino et al., 2013). A hypothesis consistent with our data and the literature is, therefore, that the LAVs are MVE precursors – similar to the endosome and lysosome vesicles that deliver MMPs to invadopodia (Poincloux et al., 2009; Pedersen et al., 2020) – and that these are the MCT4- and CD147-containing vesicles we identify here. MCT4 and CD147 would thus be retrieved from the general plasma membrane along with MMP14 and delivered to invadopodia, consistent with their increased expression in these structures. Consistent with this notion, CD147 and MCT1 have been found to be greatly enriched in MVEs released by malignant gliomas (Thakur et al., 2020). Given that MCT1 and MCT4 share their chaperone dependence on CD147, it seems reasonable to suggest that in cells such as MDA-MB-231 cells, which do not contain MCT1, MCT4 would instead be cotransported with CD147 to exosomes. This raises several new questions that are not answered in the present work. Firstly, the role of CD147 and MCT4 in matrix degradation demonstrated here does not preclude other roles of the two proteins in cancer cell invasion, such as MCT4–integrin interaction (Gallagher et al., 2009) or cytoskeletal regulation by MCT4-induced intracellular pH changes (Webb et al., 2011). Our data also do not dissect the relative importance of the roles of MCT–CD147 in LAVs versus those in invadopodia. If MCT4 plays a role in the LAVs per se, it seems likely to involve contribution to luminal acidification, in turn facilitating intraluminal cathepsin- and MMP-mediated ECM degradation (Montcourrier et al., 1994, 1990). Similarly, in invadopodia, MCT4-mediated H^+^ efflux could create a local pericellular acidic zone, enhancing protease activity. In gliomas, MCT1 and CD147 are not only enriched in exosomes but also play a causal role in their release, apparently through raising intracellular free Ca^2+^ concentrations (Thakur et al., 2020). Such possibilities, to be studied in future work, not only point to a new understanding of the role of MCT4 in cancer, but also have potential for non-invasive detection of MCT4 in cancer patients for diagnostic- or treatment-monitoring purposes.

In conclusion, we show here that MCT4 and CD147 reciprocally regulate each other and support breast cancer cell migration and invasiveness in an interdependent manner. Mechanistically, this involves an MCT4- and CD147-dependent stimulation of ECM degradation and specifically of MMP-mediated collagen-I degradation. MCT4 and CD147 colocalize strongly to invadopodial peripheral structures, as well as in large, LAMP1-positive vesicles, in both cases colocalizing with MMP14, and we suggest that these vesicles mediate delivery of MMP14 to invadopodia.

## MATERIALS AND METHODS

### Cell lines and cell culture

MDA-MB-231 and MCF10A cells were a kind gift from Dr Marie Kveiborg, Biotechnology Research Institute Copenhagen (BRIC), Copenhagen, Denmark. Cell lines were STR profile-authenticated and tested for contamination by IDEXX BioAnalytics (latest test September 2022), and were routinely tested and found negative for mycoplasma infection (approximately every 3 months). MDA-MB-231 cells were cultured in Dulbecco's modified eagle medium (DMEM) 1885 (Substrat og SterilCentralen #15, University of Copenhagen; Panum 13.01.111) supplemented with 10% fetal bovine serum (FBS; Gibco, F9665), 1% penicillin-streptomycin (Pen/Strep; Sigma-Aldrich, P0781) and 1% minimum essential medium (MEM) non-essential amino acids, 100× (NEAA; Gibco, 11140-035). For MDA-MB-231 with stable shRNA-mediated MCT4 KD (performed as described in Andersen et al., 2018) the medium was supplemented with 1 µg/ml puromycin (Gibco, A11138-02). MCF10A cells were cultured in DMEM containing 4.5 g/l D-glucose, L-glutamine and pyruvate (Gibco, 41966-029) mixed 1:1 with Nutrient Mixture F-12 (Sigma-Aldrich, N6658) supplemented with 5% FBS (Gibco, F9665), 1% Pen/Strep (Sigma-Aldrich, P0781), 20 ng/ml epidermal growth factor (EGF; Sigma, E9644), 0.5 µg/ml hydrocortisone (Sigma-Aldrich, H0888) and 1% insulin (Gibco, 41400045). Cells were cultured in culture flasks (T25 or T75; Greiner Bio-one, CELLSTAR; 690160 and 658170) at 37°C, 95% humidity and 5% CO_2_, and were passaged when a confluence of 70–80% was reached. Cell cultures were discarded when they reached passage 20–25.

### Knockdown and overexpression

#### Transient knockdown

MDA-MB-231 cells were seeded in 6-well plates (Greiner Bio-One, CELLSTAR, 657-160) and grown to a confluence of ∼25%. On the day of transfection, cells were washed once in transfection medium (MDA-MB-231 culture medium without FBS and Pen/Strep), and 1.5 ml of transfection medium was added per well. Cells were then treated with 500 µl of transfection medium containing 5 µl Lipofectamine 2000 (Invitrogen, 11668019) and the relevant siRNA, which was applied at final concentrations of 25 nM (CD147 KD) or 50 nM (MCT4 KD). At 4–6 h after transfection, 120 µl FBS was added per well. The medium was replaced with normal growth medium 24 h after transfection, and experiments were performed 48 h after transfection. The following siRNAs targeting human *SLC16A3* (encoding MCT4) or *BSG* (encoding CD147), all from Dharmacon (via VWR International A/S, Søborg, Denmark), were used (sense strand sequences are given): MCT4 si1, 5′-CCGCAAGGUUACAAGGCAUUU-3′; CD147 si1, 5′-UCCCAGUGCUUGCAAGAUUUU-3′; CD147 si2 (Dharmacon, CTM-465973), 5′-CCACCCACCGCCACAAUAAUU-3′. Mock (scrambled) siRNA was from Sigma-Aldrich, #SIC001.

#### Overexpression

MDA-MB-231 or MCF10A cells were seeded in 6-well plates (Greiner Bio-One, CELLSTAR, 657-160) and grown to a confluence of 50–70%. Prior to transfection, the culture medium in each well was replaced with 2 ml of fresh culture medium. Cells were then treated with 245 µl Opti-MEM (Gibco, 31985062) containing 2.5 µg of the relevant DNA plasmid (for dual expression, 1.25 µg of each plasmid), 7.5 µl Lipofectamine 3000 (Invitrogen, L3000-015) and 5 µl P3000 Enhancer Reagent (Invitrogen, L3000-015). After 4–6 h, the medium was replaced with fresh culture medium. Experiments were performed 24 h after transfection. The average transfection efficacy was 8.6% for MCT4 and 14.2% for CD147, estimated from immunofluorescence analysis (*n*=4). Plasmid constructs used were empty pcDNA3.1 (+) (vector control), CD147 in pcDNA3.1 (+) (GenScript, OHu27639) and rat MCT4 (rMCT4) in pcDNA3.1(+). The CD147 plasmid was a kind gift from Marie Kveiborg, BRIC, University of Copenhagen, Denmark. The rMCT4 sequence in a *Xenopu*s vector (pGHJ-rMCT4) was kindly provided by Holger M. Becker, University of Kaiserslautern, Germany. rMCT4 was subcloned from pGHJ to pcDNA3.1 (+) using restriction enzyme double digestion and sequenced before use.

### Antibodies

Primary antibodies against GAPDH [2118; western blotting (WB), 1:1000], LC3A/B [4108; immunofluorescence (IF), 1:100; WB, 1:100] and golgin-97 (97537; IF, 1:400) were purchased from Cell Signaling Technology. Primary antibodies against CD147/EMMPRIN [MAB972; WB, 1:1000; immunoprecipitation (IP), 1 mg/ml] and CD147 (AF972; IF, 1:100) were purchased from R&D Systems. Primary antibodies against MCT4 (376140; WB, 1:400; IF, 1:200; IP, 1 mg/ml) and LAMP1 (20011; IF, 1:150) were purchased from Santa Cruz Biotechnology. Primary antibodies against MMP14 (271840; WB, 1:1000 and IF, 1:100; and 192782; WB, 1:6000), p62 (56416; WB, 1:200), and phospho-cortactin (Y421) (47768; IF, 1:100) were purchased from Abcam. The primary antibody against DCTN1 (610473; WB, 1:1000) was purchased from BD Transduction Laboratories. The primary antibody against PDI (MA3-019; IF, 1:100) and Rhodamine-conjugated phalloidin (R415; IF, 1:100) were purchased from Invitrogen. The primary antibody against TOM20 (11802-1-AP; IF, 1:200) was purchased from Proteintech. The primary antibody against Src (OP07; IF, 1:100) was purchased from Calbiochem. Secondary antibodies for western blotting: horseradish peroxidase (HRP)-conjugated goat anti-mouse Ig (DAKO, P0447; either 1:2000 or 1:4000) and HRP-conjugated goat anti-rabbit Ig (DAKO, P0448; either 1:2000 or 1:4000). All secondary antibodies for immunocytochemistry were from Invitrogen, raised in donkey and used at 1:600 dilution: anti-goat IgG Alexa Fluor 488, A11055; anti-goat IgG Alexa Fluor 568, A11057; anti-goat IgG Alexa Fluor 647, A21447; anti-mouse IgG Alexa Fluor 568, A10037; anti-mouse IgG Alexa Fluor 647, A31571; anti-rabbit IgG Alexa Fluor 488, A21206; anti-rabbit IgG Alexa Fluor 568, A10042; and anti-rabbit IgG Alexa Fluor 647, A31573. For MCT4 and CD147 antibodies, specificity was confirmed by KD and OE assays. For remaining antibodies, specificity was evaluated by localization in immunofluorescence analysis and correct apparent molecular mass of bands in western blot analyses.

### SDS-PAGE and western blotting

Western blot analysis was carried out essentially as described previously (Sjøgaard-Frich et al., 2021). Briefly, cells were grown to ∼80% confluency, washed in ice-cold PBS and lysed [1% SDS, 10 mM Tris-HCl, 1 mM NaVO_3_, cOmplete Mini Protease Inhibitor (Roche, #11836153001), pH 7.5, 95°C]. Lysates were sonicated and centrifuged (21,000 ***g***, 5 min, 4°C), and protein concentrations were determined using a Bio-Rad DC Protein assay kit (Bio-Rad, #00-0113, -0114 and -0115). Protein concentrations were equalized with double-distilled H_2_O, and lysates were mixed with 1:1 NuPAGE LDS Sample Buffer 4× (Invitrogen, NP0007) and 0.5 M dithiothreitol (Sigma, 646563) then separated by SDS-PAGE using 10% Criterion TGX Precast Midi Protein Gels (Bio-Rad, 567-1035), Tris-glycine-SDS running buffer (Bio-Rad, 161-0732) and BenchMark protein ladder (Invitrogen, 10747-012). Proteins were transferred to nitrocellulose membranes (Bio-Rad, 170-4159), stained with Ponceau S (Sigma-Aldrich, 7170-1L) and blocked [1 h, 37°C, 5% dry milk in Tris-buffered saline containing 0.1% (v/v) Tween-20 (TBST)]. Membranes were washed 3× in TBST, incubated with primary antibodies in TBST with 5% BSA (Sigma, A7906) overnight at 4°C, washed in TBST, incubated with HRP-conjugated secondary antibodies for 1 h at room temperature, washed in TBST again, and developed using Clarity Western ECL Substrate (Bio-Rad, 1705061) on a Fusion FX developer (Vilber). Band intensities were quantified using ImageJ software (National Institutes of Health, Bethesda, MD, USA) and normalized to their respective loading control. The original western blots are shown as Fig. S7.

### qPCR analysis

Total RNA was isolated using NucleoSpin RNA II (Macherey-Nagel) and reverse transcribed using Superscript III Transcriptase (Invitrogen, 18080044). cDNA was amplified in triplicates utilizing SYBR Green (Roche, 04913914001) in an ABI7900 qPCR machine with the following steps: 95°C for 10 min; 40 cycles of 95°C for 30 s, 55–63°C (depending on the primer pair) for 1 min, 72°C for 30 s; then 95°C for 1 min. Primers were designed using NCBI and Primer-BLAST (www.ncbi.nlm.nih.gov), synthesized by Eurofins and diluted in nuclease-free H_2_O (2 µM working dilution). mRNA levels were determined using the Pfaffl method (Pfaffl, 2001), with housekeeping genes β-actin or TATA-binding protein (*TBP*), and are shown relative to the mRNA level in control cells. Primers used: CD147 forward, 5′-CCGTAGAAGACCTTGGCTCC-3′; CD147 reverse, 5′-TACTCTCCCCACTGGTCGTC-3′; MCT4 forward, 5′-TGCCATTGGTCTCGTGCTG-3′; MCT4 reverse, 5′-TCTGCCTTCAGGAAGTGCTC-3′; β-actin forward, 5′-AGCGAGCATCCCCCAAAGTT-3′; β-actin reverse, 5′-GGGCACGAAGGCTCATCATT-3′; TBP forward, 5′-ACCCACCAACAATTTAGTAGTTA-3′; TBP reverse, 5′-GCTCTGACTTTAGCACCTGTTA-3′.

### Co-immunoprecipitation of MCT4 and CD147

Cells transfected as above but with 7.5 µg CD147 and 3.9 µg MCT4 plasmid DNA per 10 cm Petri dish were washed in ice-cold PBS and lysed in room temperature NP40 lysis buffer [50 mM Tris-HCl pH 7.4, 140 mM NaCl, 3 mM Na_3_VO_4_, 1% v/v IGEPAL CA-360 (Sigma, I8896), phosphatase inhibitor cocktail (PhosStop) and protease inhibitor mix (cOmplete) (Roche, 04906845001 and 1169748001, respectively)]. Lysates were homogenized using a 0.5 mm syringe needle and normalized to a protein concentration of ∼1.7 mg/ml in 600 μl. Samples were incubated overnight at 4°C with primary antibodies (goat anti-CD147, MAB972, R&D Systems; mouse anti-MCT4, 376140, Santa Cruz biotechnology) rotating end-over-end. For equilibration, Dynabeads Protein G (Invitrogen, 10004D) were washed twice for 10 min at 4°C with lysis buffer while gently rotating, and immune complexes were incubated with 1.5 mg washed Dynabeads for 45 min at 4°C rotating end-over-end. Dynabeads with bound protein were washed five times for 5 min each in lysis buffer, boiled for 5 min at 95°C in 80 μl NuPAGE LDS Sample Buffer (Invitrogen, NP0007) and dithiothreitol, vortexed thoroughly, and placed on ice for 30 min. Eluted protein complexes were separated using SDS-PAGE and analyzed by western blotting as described above.

### Immunofluorescence microscopy analysis and line scan quantifications

Cells were grown on glass coverslips that, if so specified, were pre-coated with Oregon Green 488-conjugated gelatin (Invitrogen, G13186). Cells were washed in ice-cold PBS, fixed in 2% paraformaldehyde (PFA; Sigma, 47608), permeabilized in 0.5% Triton X-100 (Sigma, X100) in TBST for 5 min, washed twice for 5 min in TBST and blocked in 5% BSA (Sigma, A7906) in TBST for 30 min. Coverslips were incubated overnight at 4°C with primary antibodies in 1% BSA in TBST, washed three times for 5 min each in 1% BSA in TBST, incubated with Alexa Fluor-conjugated secondary antibodies for 1 h at room temperature and then washed in 1% BSA in TBST for 5×5 min, with 4′,6-diamidino-2-phenylindole (DAPI; Invitrogen, D3571) at 1:1000 in 1% BSA included in the second wash. For LAMP1 staining, 0.5% saponin (Sigma, 47036) was additionally present at all steps in the protocol where BSA was used. Coverslips were mounted using 2% N-propyl gallate (Sigma, P-3130) and sealed with nail polish, and proteins were visualized with an Olympus IX83 microscope with a Yokogawa spinning disc, using confocal imaging, a 60×/1.4 NA oil immersion objective and CellSens Dimension software (Olympus). Image overlays, intensity adjustments and maximal-intensity *z*-projections were carried out in ImageJ software. Where indicated, *z*-stack deconvolution was carried out using CellSens software.

Plasma membrane localization was quantified using the ImageJ Color Profiler plugin. All analyses were performed with the experimental conditions masked to avoid any bias. For each condition, 4–6 images were analyzed. A line selection was drawn across the plasma membrane. The Color Profiler tool was used to create a plot showing distance along the selected line on the *x*-axis and gray values (pixel intensity) on the *y*-axis. The ‘List’-option outputs a list with all pixel intensities. From each line scan, the maximum intensity value was used to calculate the mean value for each individual image (11–21 cells per image). Experimental conditions were unmasked, and a mean intensity value was calculated for each condition.

### Flow cytometry

Cells transfected as described above were detached, resuspended in growth medium, spun down at 80 ***g*** for 3 min and resuspended in flow buffer (PBS containing 2% FBS and 0.1% sodium azide). A million cells per condition were then stained with PE-conjugated anti-human CD147 antibody diluted 1:1000 in flow buffer (BioLegend, 306211) for 20 min on ice. A sample with a million vector-transfected cells incubated in flow buffer without antibody was included as an unstained control sample. Cells were subsequently fixed in fix buffer (PBS containing 2% FBS and 1% PFA) for 20 min on ice, resuspended in flow buffer and stored at 4°C until analyzed on a BD FACSCalibur cytometer, followed by subsequent data analysis using FlowLogic v. 8.6 (Inivai Technologies). The following gating strategy was employed: cells of interest were selected based on a forward scatter (FSC) versus side scatter (SSC) plot, and a gate marking the CD147-negative population was defined based on the unstained vector control sample. The stained vector control sample was used to define the gates containing the CD147-positive population and the CD147-OE population. Results are shown as percentage of cells in specified gates.

For flow cytometry analyses performed simultaneously with migration and invasion assays, cells were trypsinized and spun down at 100 ***g*** for 3 min, resuspended in PBS containing 1% FBS and spun down. Cells were then resuspended in PBS with 1% FBS and fixed in 96% ethanol. Fixed cells were resuspended in ice-cold PBS containing 5 μg/ml propidium iodide (PI; Invitrogen, P3566) and 5 μg/ml DNase-free RNase-A (Sigma-Aldrich, 11119915001) and incubated at 37°C for 30 min, protected from light. The samples were transferred to polystyrene tubes (BD Falcon, 352038) and a negative sample treated only with RNase was included to control for autofluorescence. Cell cycle distribution was determined by flow cytometry analysis of nuclear DNA content using a FACSCalibur flow cytometer and CellQuest software (BD Biosciences).

### Transwell migration and invasion assays

Migration and invasion were analyzed using 24-well 8 µm control inserts (Corning, 354578) and Matrigel-coated invasion inserts (Corning, 354483), respectively. Before seeding, cells were briefly starved in low serum medium (1% FBS, 1% NEAA). In most experiments, 50,000 cells per chamber were seeded onto the inserts in medium containing 1% FBS. Medium containing 10% FBS was added to the lower chambers, and chambers were incubated at 37°C and 5% CO_2_ for 20–24 h. After washing with Gurr buffer (Gibco, 10582013), non-migrating/invading cells were gently removed using a cotton swab. The cells located on the lower side of the chamber were fixed in ice-cold absolute methanol for 1 h, washed twice in Gurr buffer and stained with 30% Giemsa stain solution (Sigma-Aldrich, 51811-82-6) for 30 min. Membranes were washed three times in Gurr buffer and air-dried. Alternatively, 10,000, 25,000 and 45,000 cells were seeded per chamber, and cells were fixed in 4% PFA for 20 min at room temperature, permeabilized in 0.5% Triton-X100 in TBST for 20 min and stained with DAPI (1:1000) for 10 min at room temperature. All other procedures were the same as above. Stained membranes were cut out and placed on a glass slide. Membranes were imaged using a 40×/0.9 NA air immersion objective and bright-field illumination on an Olympus IX83 microscope. Cells were counted manually from 10–20 images per membrane. To relate migration and invasion to proliferation, bromodeoxyuridine (BrdU) assays were performed simultaneously with migration and invasion assays. Cells were seeded in 96-well plates in triplicates at 3×10^3^ and 10×10^3^ cells per well. After 24 h, BrdU labeling reagent was added (1:100; Roche, 11669915001). After 4 h, cells were fixed and stained with BrdU antibody according to the manufacturer's protocol (Roche, 11669915001). BrdU incorporation was measured using a FLUOstar OPTIMA microplate reader (BMG Labtech).

### Quantification of gelatin degradation

Glass coverslips were coated with 60°C preheated Oregon Green 488-conjugated gelatin from pig skin (Invitrogen, G13186) at 0.5 mg/ml in PBS containing 2% sucrose (VWR, 27480.294). Coverslips were placed on a piece of Parafilm, and 45 µl gelatin was added to a coverslip and transferred to the next coverslip by tilting the coverslip vertically with tweezers. To form a thin uniform layer, excess gelatin was removed by gentle aspiration from the periphery of the coverslip using a vacuum source. The gelatin from the second coverslip was transferred back to the Eppendorf tube kept at 60°C, and the procedure was repeated for the remaining coverslips. Coated coverslips were placed in a 12-well plate protected from light and left to dry for ∼1–1.5 h. For fixation, each well was incubated for 15 min on ice with 1 ml of pre-chilled 0.5% glutaraldehyde (Sigma, G6257) diluted in PBS, followed by three washes in PBS at room temperature. Each well was incubated with 1 ml of freshly prepared 5 mg/ml sodium borohydride (Sigma-Aldrich, 452882) in PBS for 3 min followed by three washes in PBS. Coverslips were stored in 12-well plates in PBS with 1% Pen/Strep at 4°C until use. Cells were seeded on the gelatin-coated coverslips in medium without Pen/Strep at 70,000 cells per ml. After 4, 8 or 12 h, as indicated, coverslips were washed in PBS, fixed in 4% PFA (Sigma, 47608) in PBS for 10 min at room temperature, followed by three washes in PBS. Cells were stained according to the IFM procedure described above using Rhodamine-conjugated phalloidin (RP; Invitrogen, R415) to visualize F-actin and DAPI to visualize nuclei. Images were obtained employing an Olympus IX83 microscope. The degraded area fraction was quantified using the AdaptiveThreshold plugin and the Area Fraction measurement from the Measure tool in ImageJ. Cell counts were obtained by manually counting the nuclei using the multi-point tool in ImageJ. For each image, the degraded area fraction was divided by the cell count. The relative degradation for a given time point and condition was then calculated as the mean of measurements from 6–13 images.

### Collagen-I cleavage analysis

The wells of 6-well plates were coated with collagen-I by adding 1 ml of 50 µg/ml collagen type I (Corning, 354249) in 20 mM acetic acid to each well, followed by 1 h 20 min incubation at 37°C. Remaining collagen solution was removed, and wells were rinsed three times with PBS heated to 37°C. The plate was left to air dry in the cell culture hood for 1 h, then wrapped in parafilm and stored at 4°C until cells were seeded. Transfected MDA-MB-231 cells were resuspended in culture medium without Pen/Strep and seeded on collagen-coated 6-well plates at a density of 350,000 cells per well. After 48 h, 1 ml of culture medium was sampled from each condition and stored at −20°C. reC1M (matrix metalloproteinase-generated fragments of type I collagen) concentrations were determined using a competitive ELISA according to the manufacturer's instructions (1000AG01; Nordic Bioscience, Herlev, Denmark). In brief, 96-well streptavidin-coated plates were coated with a biotinylated synthetic reC1M peptide and incubated for 30 min at 20°C. A calibrator reC1M peptide or relevant samples were added to designated wells followed by the addition of peroxidase-conjugated specific monoclonal antibodies. The plate was incubated overnight at 4°C before tetramethylbenzidine was added to each well and the plates were incubated again for 15 min at 20°C. All incubation steps included shaking at 300 rpm, and plates were washed five times with wash buffer (20 mM Tris-HCl, 50 mM NaCl, pH 7.2) after each incubation. Lastly, the reaction was stopped by adding 0.18 M H_2_SO_4_, and absorbance was measured at 450 nm, with 650 nm as reference.

### Transfection with Src Y527F plasmid

For transfection with DNA plasmids, MDA-MB-231 cells were seeded in 6-well plates (Greiner Bio-One, CELLSTAR, 657-160) and grown to a confluence of 50–70%. Prior to transfection, the culture medium in each well was replaced with 2 ml of fresh culture medium. Cells were then treated with 245 μl Opti-MEM (Gibco, 31985062) containing 1 μg of the DNA plasmid, Lipofectamine 3000 (Invitrogen, L3000-015; 3 μl per well) and P3000 Enhancer Reagent (Invitrogen, #L3000-015; 2 μl per well). Medium was replaced with fresh culture medium after 4–6 h, and experiments performed after 24 h. The Src Y527F plasmid was pBK-CMV containing the chicken Src sequence with the Y527F mutation that renders the resulting Src protein constitutively active. The plasmid was kindly provided by Marie Kveiborg, BRIC, Copenhagen, Denmark.

### Live imaging of cells growing on Oregon Green 488-conjugated gelatin

0.5 mg/ml Oregon Green 488-conjugated gelatin (Invitrogen, G13186) in PBS with 2% sucrose (VWR, 27480.294) was preheated to 60°C. A μ-Slide 4-well plate (Ibidi, 80426) was heated to 60°C and coated with 10–30 μl of gelatin in each well. In some wells, gentle aspiration was used to remove excess gelatin in the corners of the wells to try to optimize the quality of the gelatin. The plate was left to dry in a flow bench, without a lid and protected from light, for ∼4 h. Subsequently, the gelatin-coated wells were treated with 0.5% glutaraldehyde and 5 mg/ml sodium borohydride solutions and washed several times, using volumes of 700 μl per well.

MDA-MB-231 cells were trypsinized, spun down at 150 ***g*** for 3 min, and resuspended in Phenol Red-free DMEM imaging medium (Gibco, 21063-029) supplemented with 10% FBS (Gibco, F9565), 1% Pen/Strep (Sigma-Aldrich, P0781), 1% NEAA (Gibco, 11140-035), and 1% sodium pyruvate (100 mM; Gibco, 11360-039). Cells were seeded in the gelatin-coated wells (57,000 cells in 700 μl per well) and grown for 3–4 h at 37°C to allow attachment. Fresh imaging medium was added to the wells, and the 4-well plate was placed in the microscope for image acquisition. Images were acquired every 15 min for 14 h employing an Olympus IX83 microscope with a Yokogawa spinning disc, using bright-field illumination and the 488 nm confocal laser, 40× objective and CellSens Dimension software. To enable live-cell imaging, the microscope was set up to ensure proper conditions for cell culturing (5% CO_2_, 37°C and connection to a humidifier).

### Data analysis and statistics

Data analysis and illustrations were performed using Microsoft Excel, GraphPad Prism, ImageJ and BioRender software. Data is shown as representative images or as means with s.e.m. error bars, and represent at least three independent experiments, unless stated otherwise. Statistical analyses were performed in GraphPad Prism version 8.4.1. To test for statistical differences between two groups, a two-tailed *t*-test was performed. One-way analysis of variance (ANOVA) with Dunnett's multiple comparison post-hoc test was used when more than two groups were compared. Experiments where cells were seeded from the same culture flask and transiently transfected prior to the experiment were regarded as paired.

## Supplementary Material



10.1242/joces.261608_sup1 Supplementary information

## Data Availability

All relevant data can be found within the article and its supplementary information.
