# Supplementary material for: MCT4 and CD147 colocalize with MMP14 in invadopodia and support matrix degradation and invasion by breast cancer cells
Source: J Cell Sci. 2024 Apr 30;137(8):jcs261608. doi: 10.1242/jcs.261608 (PMC11112124; doi:10.1242/jcs.261608)
Supplement: Supplementary information [file joces-137-261608-s1.pdf]

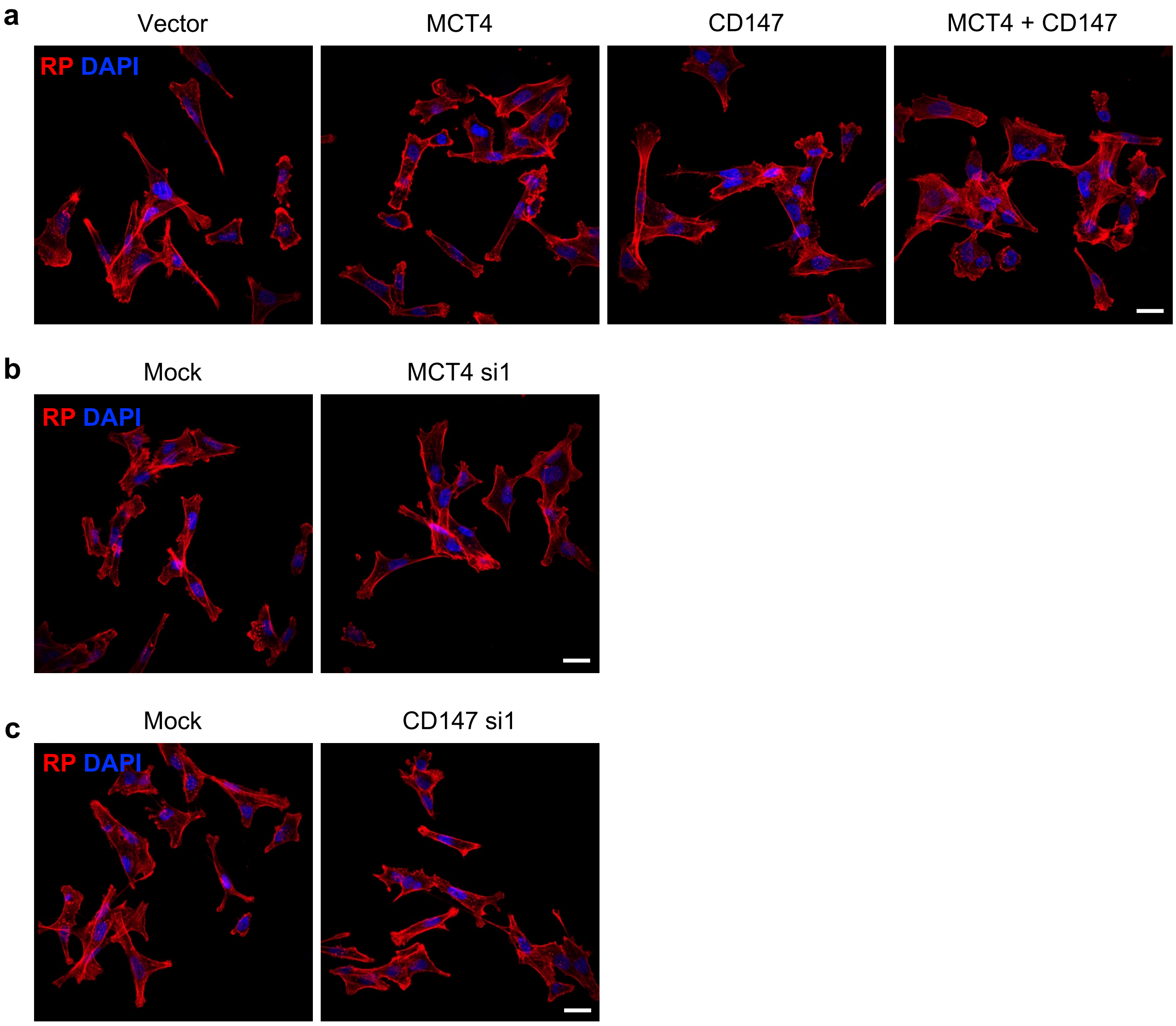

Fig. S1. Morphology of MDA-MB-231 cells was not detectably altered upon knockdown or overexpression of MCT4 and/or CD147

MDA-MB-231 cells transfected with MCT4, CD147 or MCT4 and CD147 plasmids or empty pcDNA3.1 (+) vector as negative control **(a)** or with siRNA targeting MCT4 **(b)** or CD147 **(c)** or control scrambled siRNA (mock) were seeded on gelatin-coated coverslips and grown for 12 h. Cells were subsequently subjected to ICC analysis of F-actin (Rhodamine Phalloidin, RP), and nuclei were stained with DAPI. The figure shows representative images of cells grown on gelatin for 12 h presented as merged images of rhodamine-phalloidin staining of F-actin (RP, red) and DAPI (blue). Scale bars: 20 µm. n = 3 independent experiments.

Journal of Cell Science • Supplementary information

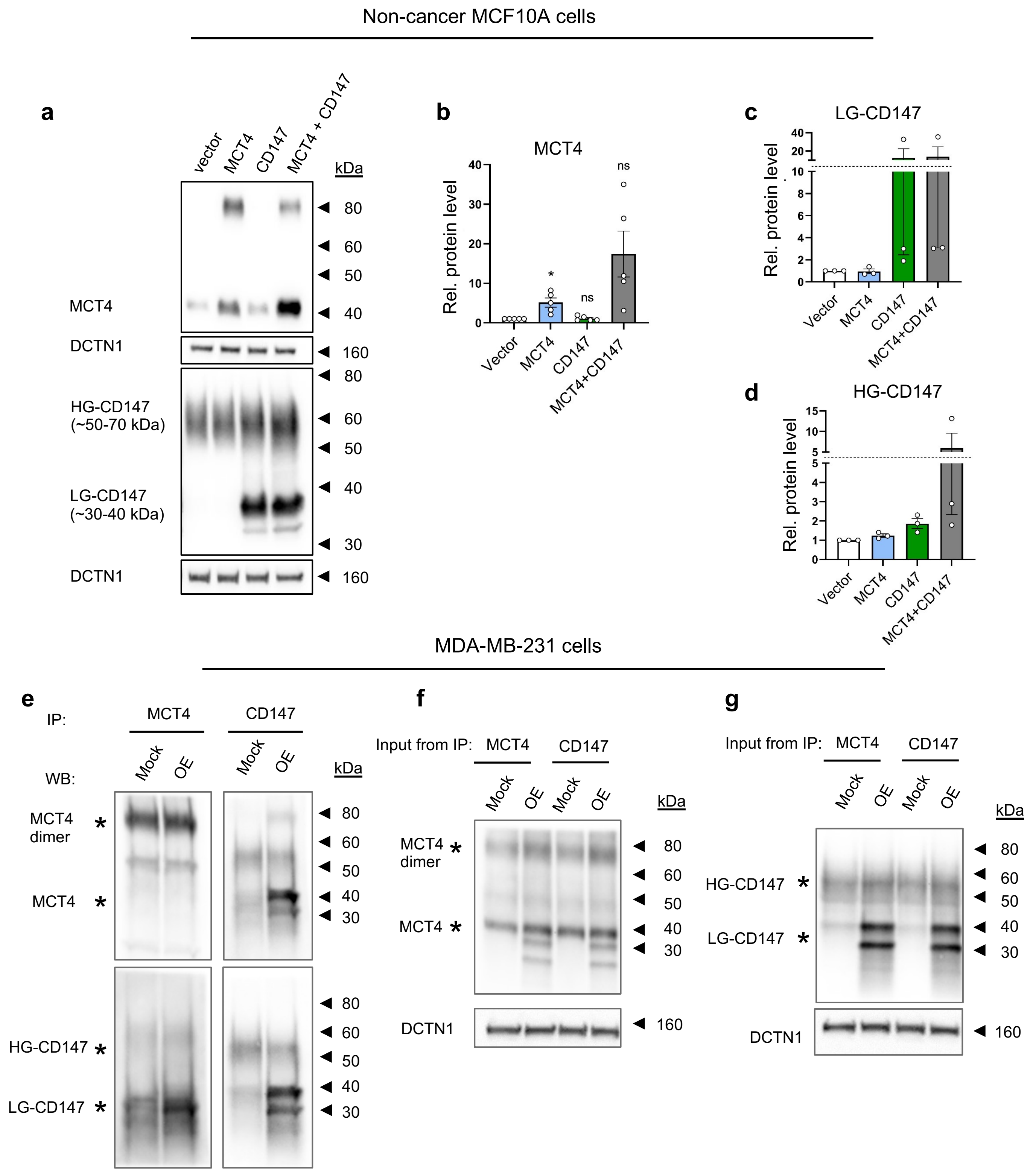

Journal of Cell Science • Supplementary information

Fig. S2. MCT4 and CD147 expression in MCF10A cells; MCT4 and CD147 co-IP in MDA-MB-231 cells

(**a**) Western blots and corresponding quantification of MCT4 and CD147 protein levels in MCF10A cells. Cells were transfected with MCT4, CD147, both, or empty vector. 24 h later, cells were lysed and subjected to Western blotting with DCTN1 as loading control. (**b-d**) MCT4 (b), LG-CD147 (c) and HG-CD147 (d) band intensities were normalized to loading controls and to vector control. Error bars denote SEM. n=5 (MCT4), 3 (CD147). Statistics: One-way ANOVA with Dunnett's post-test. *p<0.05, ns: non-significant. **(e-g)** MCT4 and CD147 co-immunoprecipitate (co-IP). MDA-MB-231 cells were transfected or not with MCT4 and CD147 as above, subjected to co-IP, and analysed by Western blot. (e) IP using antibody against MCT4 (left) and CD147 (right), (f-g) Input fraction blotted for MCT4 (f) and CD147 (g), with DCTN1 as loading ctrl. n=3 for all conditions.

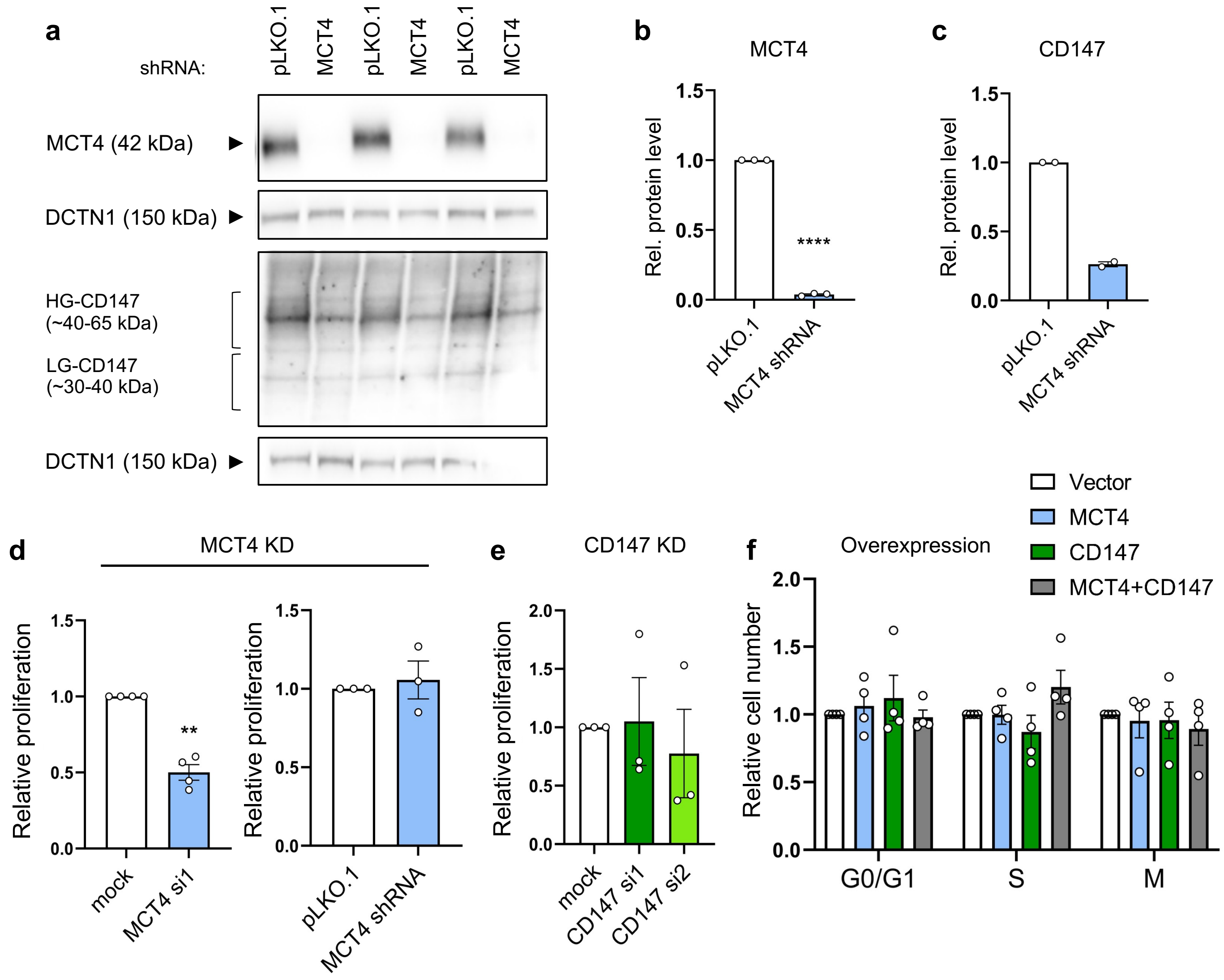

Fig. S3. Controls. Stable KD of MCT4, and BrdU and flow cytometry data

a-c. MCT4 stable KD and pLKO.1 control MDA-MB-231 cells were lysed and subjected to Western blot analysis. **(a)** Western blot showing MCT4 (top) and CD147 (bottom) protein expression. **(b-c)** Quantification of MCT4 (b) and CD147 (c) protein levels. DCTN1 was used as loading control. MCT4 and CD147 band intensities were normalized to their respective loading controls and control (pLKO.1). Error bars denote SEM. n=2. Statistics: Two-tailed paired t-test. **** denotes p<0.0001. MDA-MB-231 cells were transfected with siRNA targeting MCT4 **(d, left)** or were stably transfected with MCT4 shRNA (**d, right**) or CD147 (**e**) or scrambled siRNA control (mock), or with MCT4, CD147, or both plasmid constructs (**f**) or ctrl. vector. After 24 h (overexpression, OE) or 48 h (KD), transfected cells and MDA-MB-231 cells with stable MCT4 KD (**d, right**) were resuspended and seeded for BrdU assay (KD) or flow cytometry (OE), in parallel with migration and invasion assays. **(d-e)** Relative proliferation of siRNA-treated and stable MCT4 KD MDA-MB-231 cells as shown. 3×10^3 and 10×10^3 cells were seeded in triplicates in 96-well plates. After 24 h, cells were fixed and BrdU incorporation was measured. Values were normalized to their respective control (mock or pLKO.1). Data in d-e is the average of the values for 3×10^3 and 10×10^3 cells. Error bars denote SEM. n=4 (MCT4 siRNA KD), or 3 (MCT4 stable KD, CD147 siRNA KD). **(f)** Flow cytometry analysis of MDA-MB-231 cells overexpressing MCT4, CD147 or both, or control vector, stained with Propidium Iodide. The graph shows the relative cell number in the cell cycle stages G0/G1, S and M, normalized to vector control. Error bars denote SEM. n= 4. Statistics: Two-tailed, paired Students *t*-test (a). **: p<0.01.

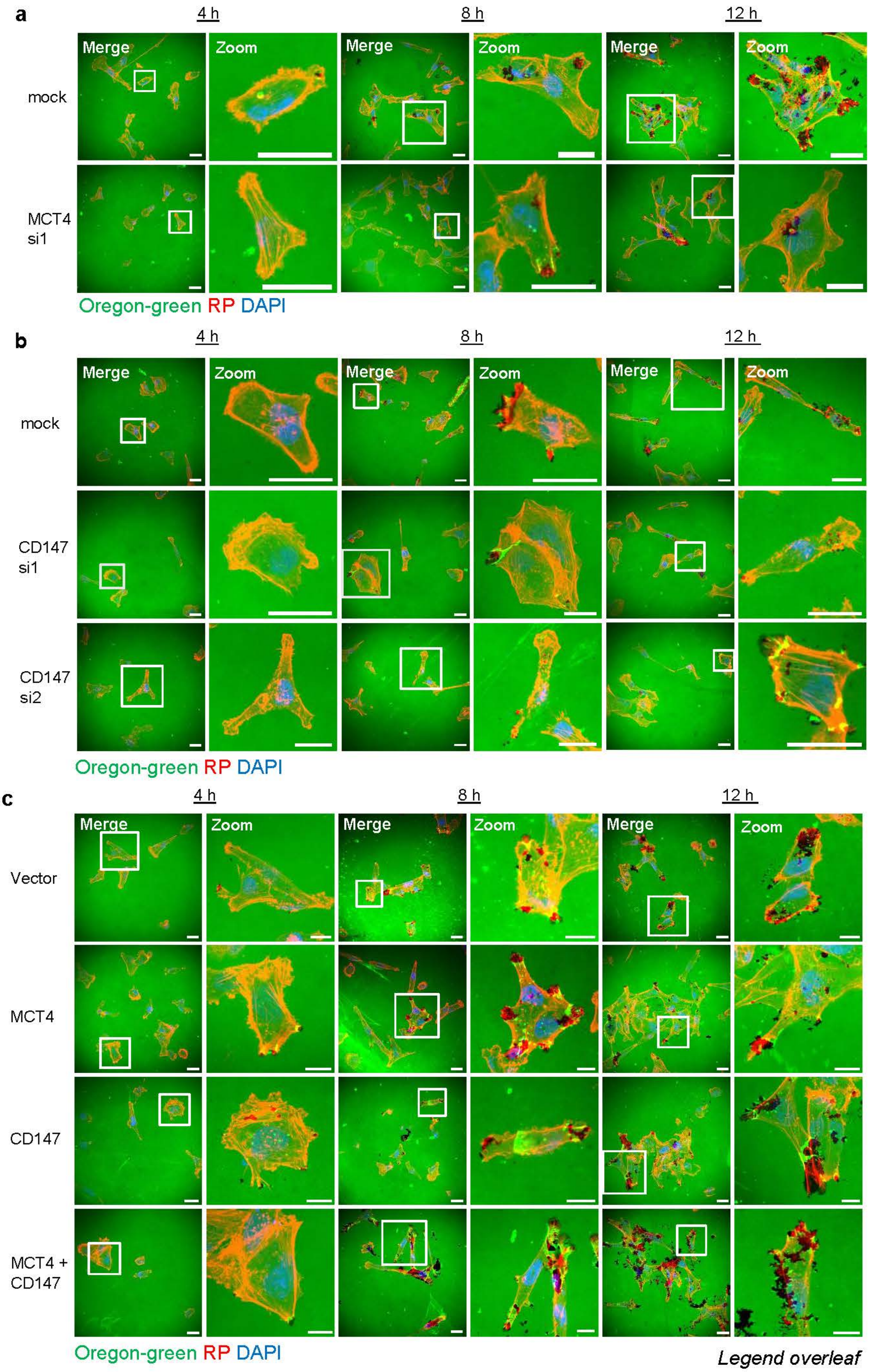

Legend overleaf

Journal of Cell Science • Supplementary information

Fig. S4. Gelatin degradation assay

(a, b) MDA-MB-231 cells transfected with siRNA targeting MCT4 (a) or CD147 (b) or control scrambled siRNA (mock) were seeded on Oregon-green conjugated gelatin-coated coverslips and grown for 4, 8 or 12 h. Cells were subsequently subjected to ICC analysis of F-actin (Rhodamine Phalloidin, RP), and nuclei were stained with DAPI. Representative images of cells grown on gelatin for the indicated time points are shown, presented as merges of gelatin (green), F-actin (RP, red) and DAPI (blue). For each time point, the right column shows zooms. Scale bars: 20 µm. n = 4-6.

(c) MDA-MB-231 cells transfected with MCT4, CD147 or MCT4 and CD147 plasmids or empty pcDNA3.1 (+) vector as negative control were seeded on oregon-green conjugated gelatin-coated coverslips and grown for 4, 8 or 12 h. Cells were subsequently subjected to ICC analysis of F-actin (Rhodamine Phalloidin, RP), and nuclei were stained with DAPI. Representative images of cells grown on gelatin for the indicated time points are shown, presented as merges of gelatin (green), F-actin (RP, red) and DAPI (blue). For each time point, the right column shows zooms. Scale bars: 20 µm, 10 µm for zooms. n = 5-7.

Journal of Cell Science • Supplementary information

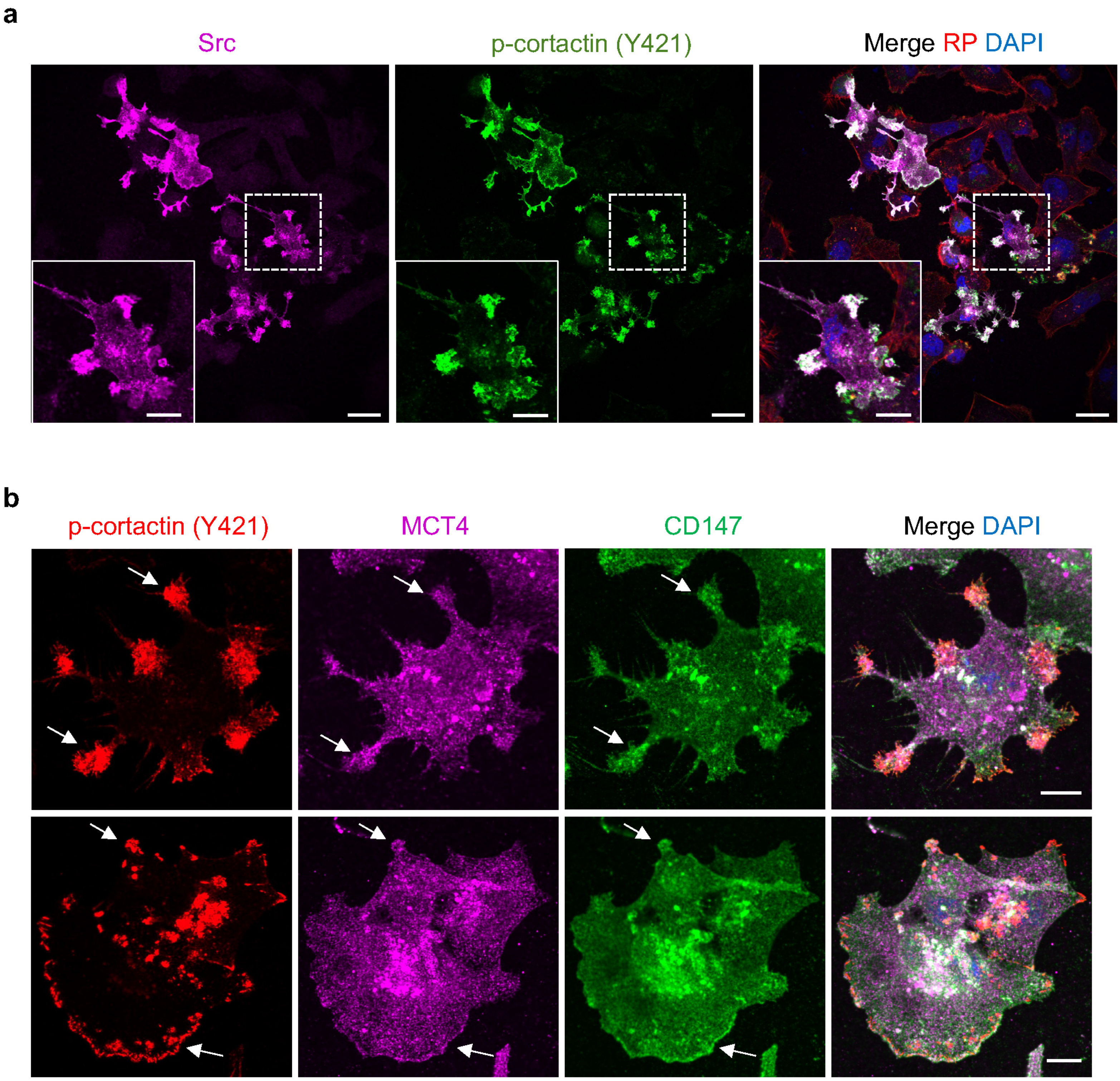
a
Src
p-cortactin (Y421)
Merge RP DAPI
b
p-cortactin (Y421)
MCT4
CD147
Merge DAPI

Fig. S5. MCT4, CD147, and p-cortactin in Y527F-Src-transfected MDA-MB-231 cells MDA-MB-231 cells were seeded on glass coverslips and transfected with a constitutive active Y527F-Src plasmid construct. 24 h post transfection cells were fixed and subjected to ICC analysis using primary antibodies targeting Src (magenta), pY421-cortactin (green) and the F-actin probe, RP (red) in (a) or MCT4 (magenta), CD147 (green) and pY421-cortactin (red) in (b). DAPI was used to visualize nuclei. **(a)** Representative image showing Src and pY421-cortactin co-localization in Y527F-Src-transfected cells. Scale bars: 20 µm or 10 µm (zooms) n=1. **(b)** Representative zooms showing the localization of MCT4, CD147 and pY421-cortactin in Y527F-Src-transfected cells. White and blue arrows indicate an overlap of pY421-cortactin, MCT4 and CD147 staining appearing as dense round structures (white) or lamellipodia-like structures (blue). Yellow arrows indicate pY421-cortactin-rich structures showing no clear overlap with MCT4 and CD147 staining and grey arrows indicate membrane co-localization of MCT4 and CD147 in a cell with no pY421- cortactin staining. Scale bars: 10 µm. n=2.

Journal of Cell Science • Supplementary information

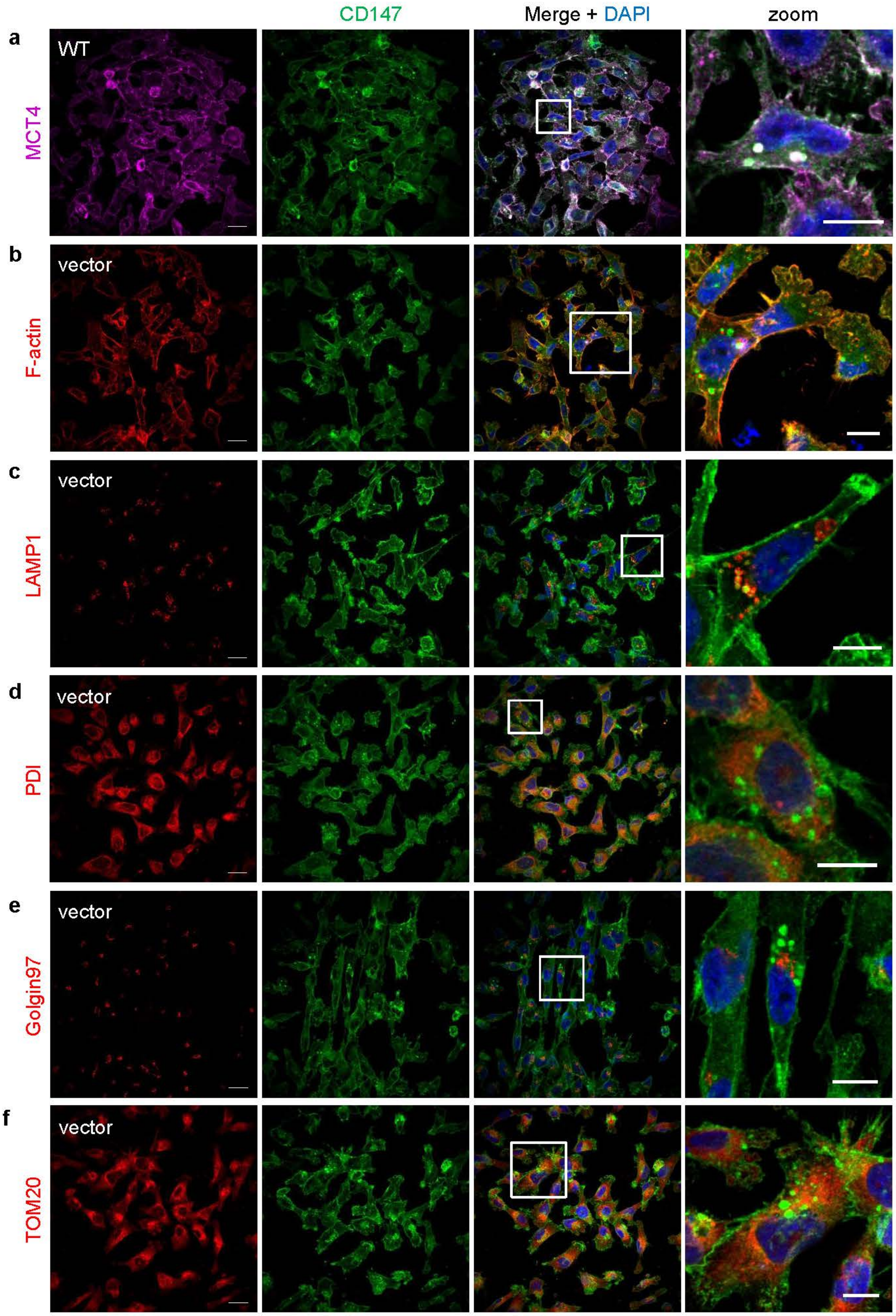

Legend overleaf

Fig. S6. Identity of intracellular structures in MDA-MB-231 cells

WT MDA-MB-231 cells (a) or MDA-MB-231 cells transfected with empty pcDNA3.1 (+) vector (b-f) were subjected to ICC analysis (24 h post transfection) using primary antibodies targeting CD147 (green) **(a-f)** in combination with either an F-actin probe, RP, (red) **(b)**, or with primary antibodies targeting MCT4 (magenta) **(a)**, LAMP-1 (red) **(c)**, PDI (red) **(d)**, Golgin97 (red) **(e)** or TOM20 (red) **(f)**. Scale bars: 20 μm or 10 μm (zooms). Images are representative of 1 n.

Journal of Cell Science • Supplementary information

Fig 1a and 1c

Unexposed (white light)

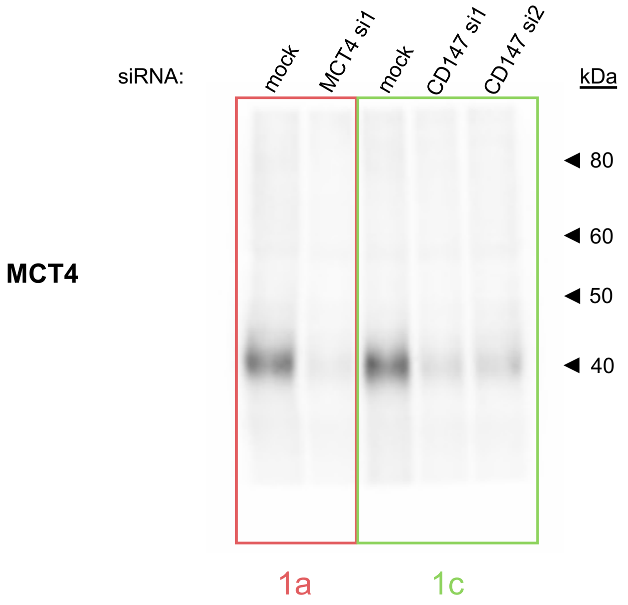

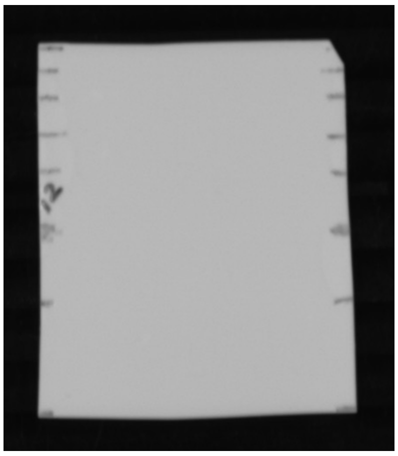

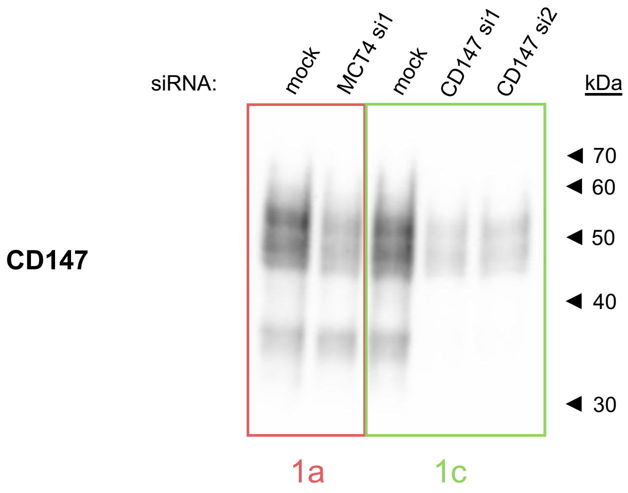

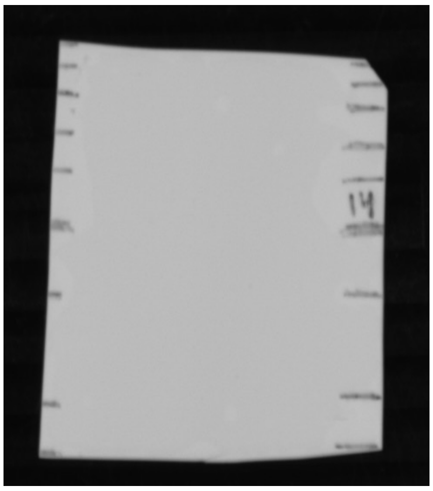

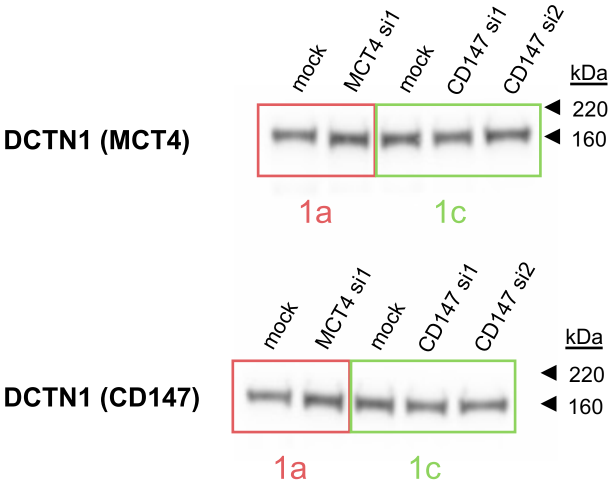

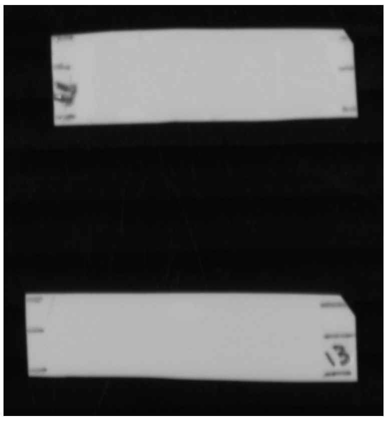

Journal of Cell Science • Supplementary information

Fig 1e

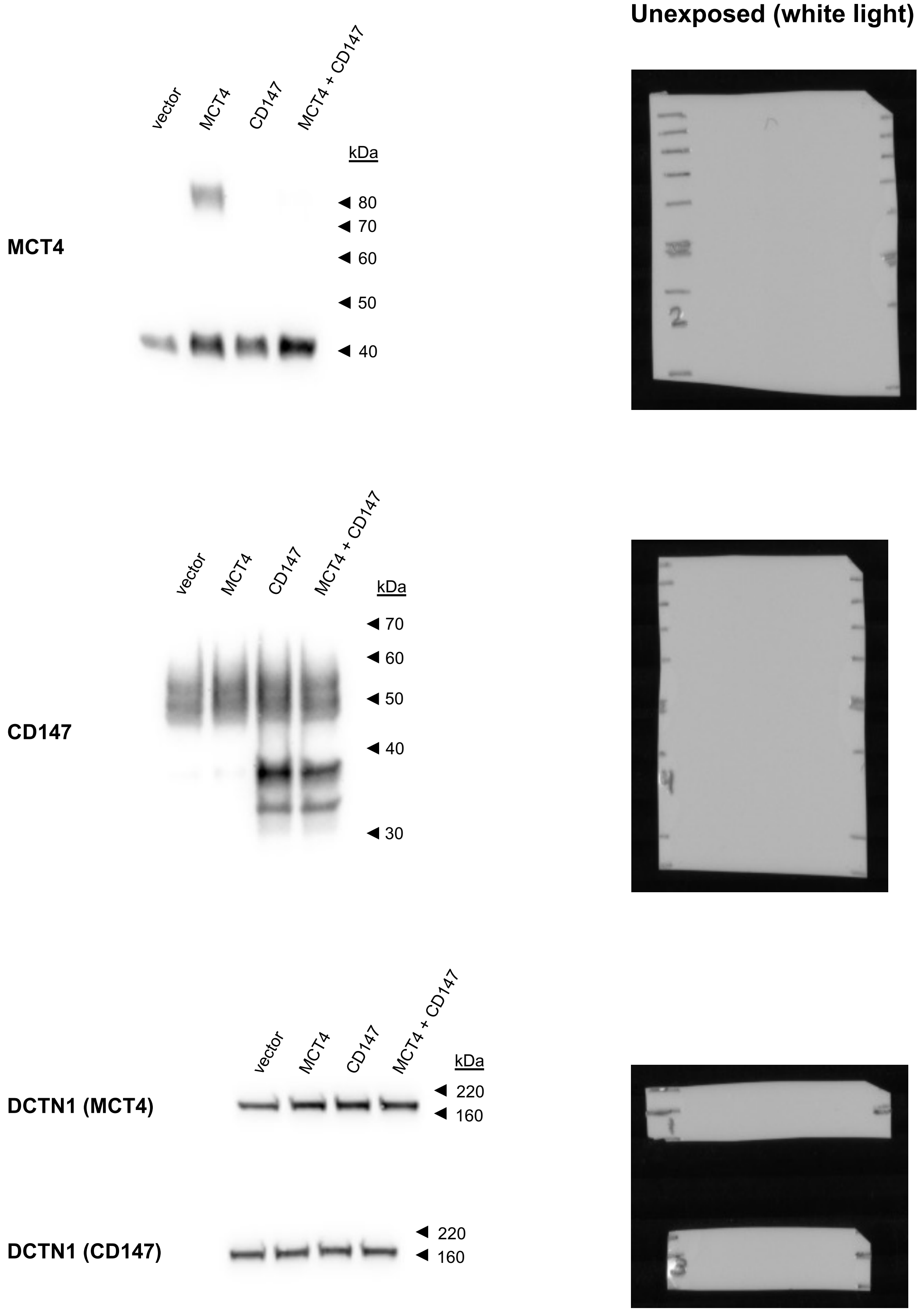
Unexposed (white light)
vector
MCT4
CD147
MCT4 + CD147
kDa
80
70
60
50
40
MCT4
vector
MCT4
CD147
MCT4 + CD147
kDa
70
60
50
40
30
CD147
vector
MCT4
CD147
MCT4 + CD147
kDa
220
160
DCTN1 (MCT4)
220
160
DCTN1 (CD147)

Fig 5c

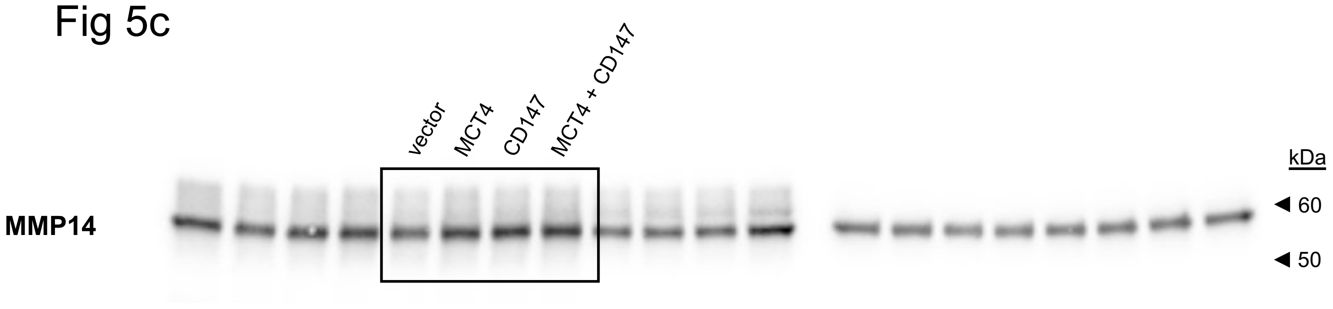

Unexposed (white light)

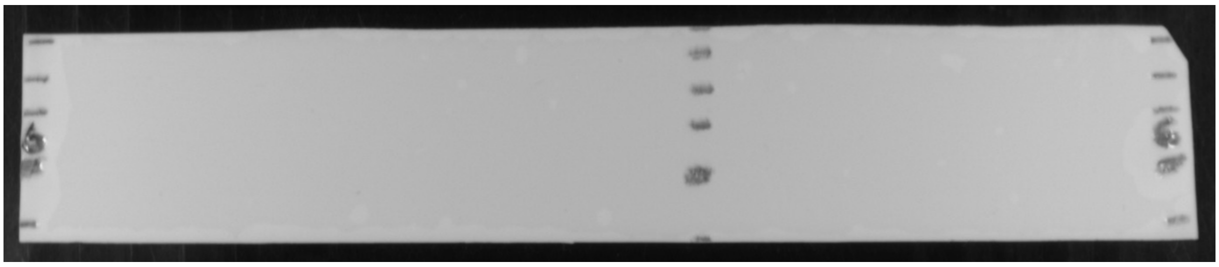

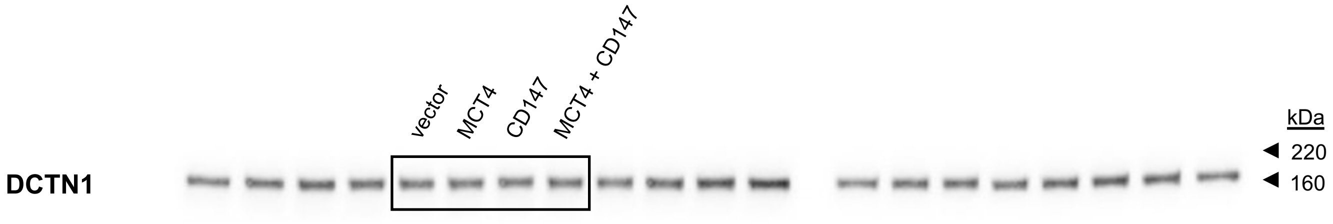

Unexposed (white light)

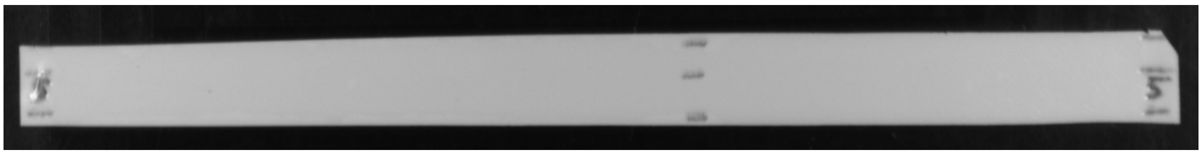

Journal of Cell Science • Supplementary information

Fig 6d

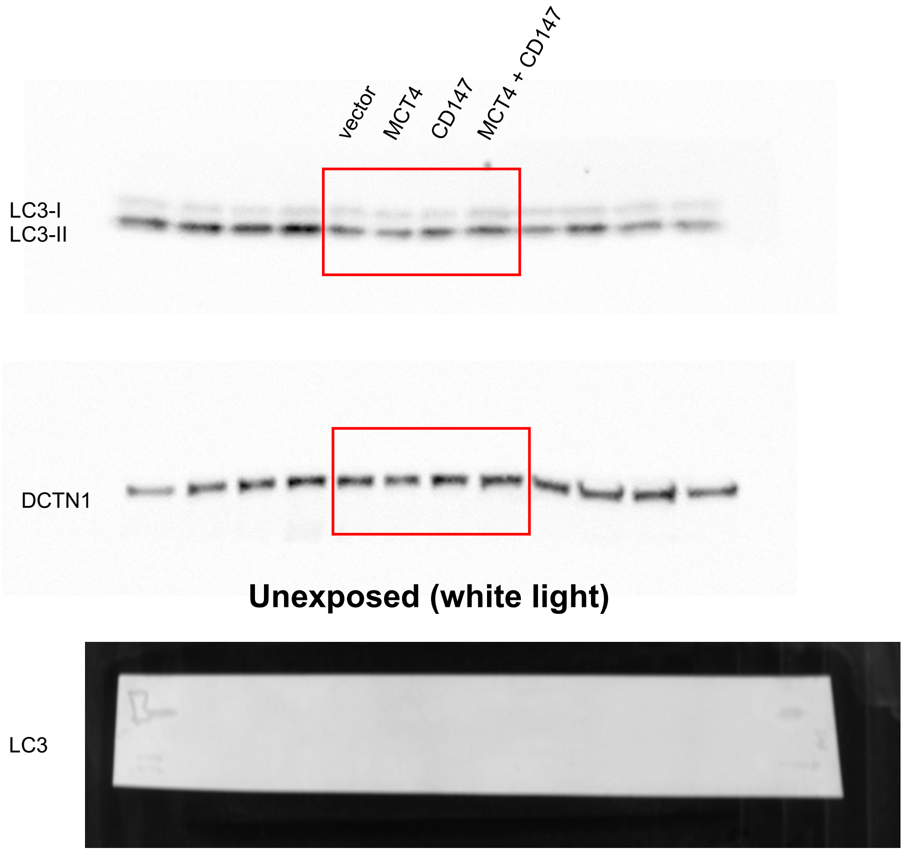

Fig 6f

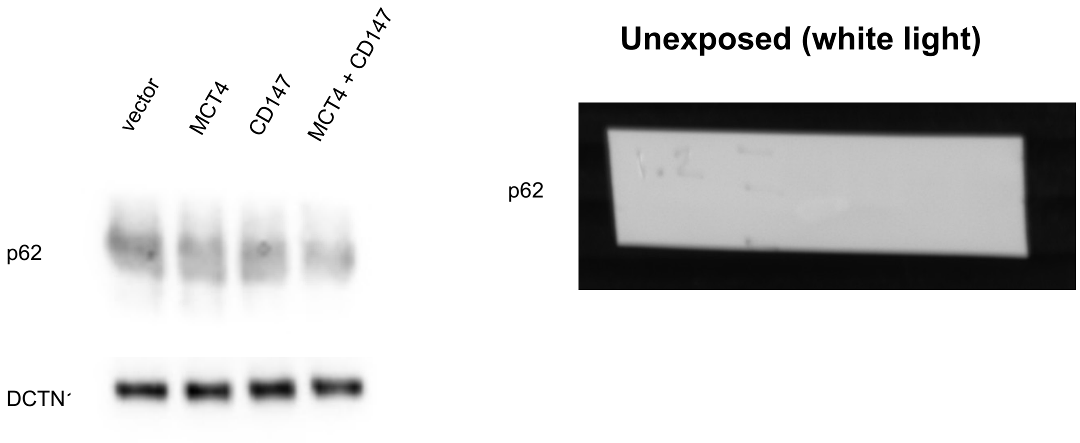

Journal of Cell Science • Supplementary information

Suppl. fig 2a

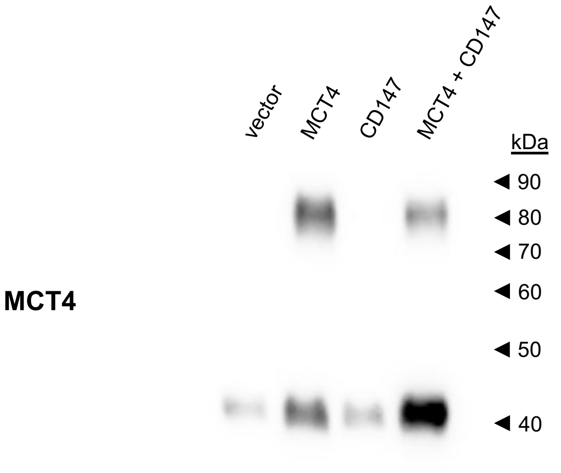

Unexposed (white light)

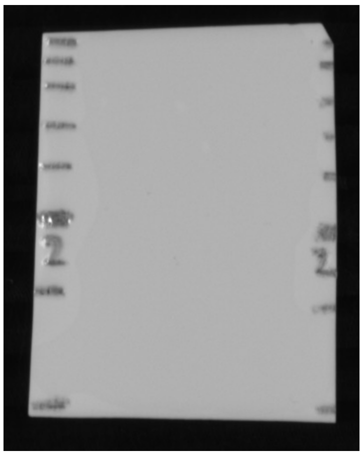

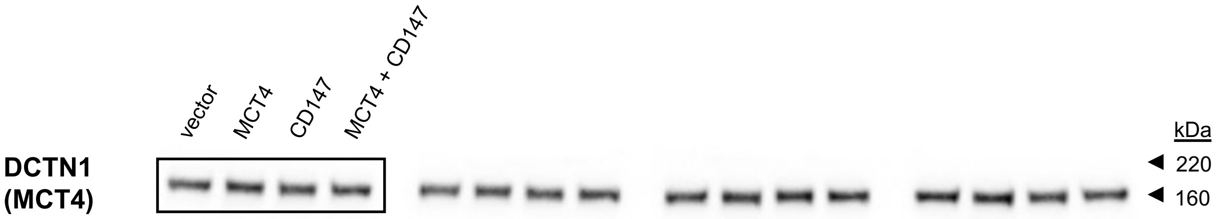

Unexposed (white light)

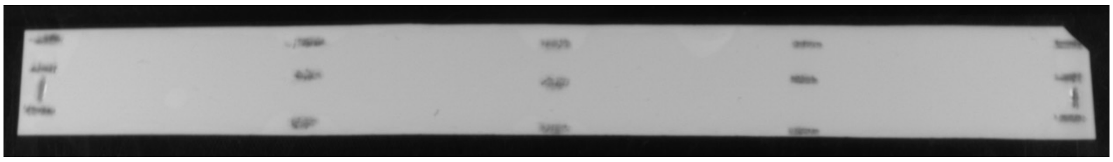

Journal of Cell Science • Supplementary information

Suppl. fig 2a continued

CD147

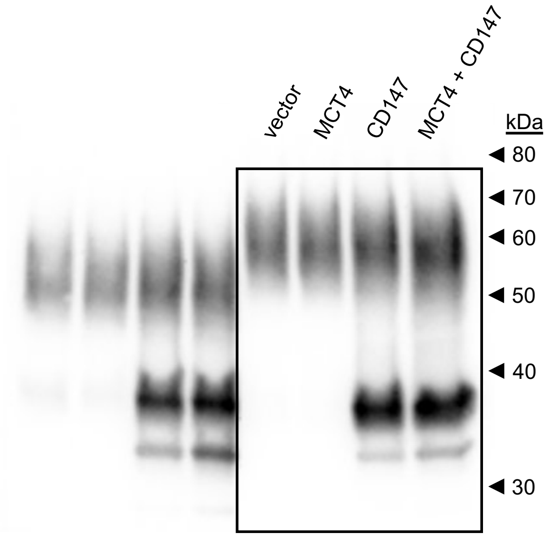

Unexposed (white light)

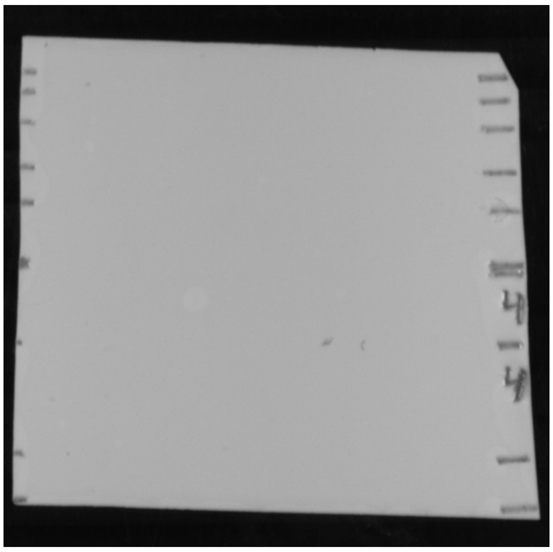

DCTN1 (CD147)

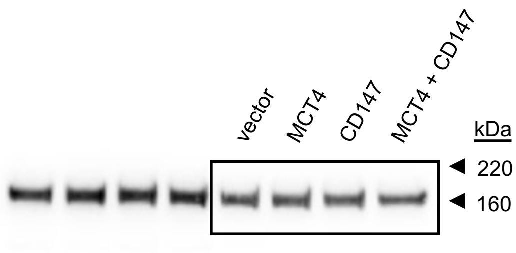

Unexposed (white light)

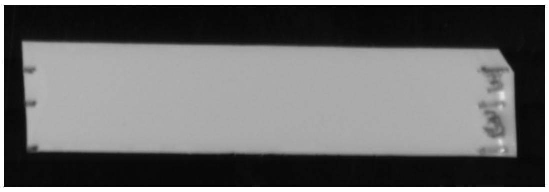

Suppl. fig 3a

MCT4

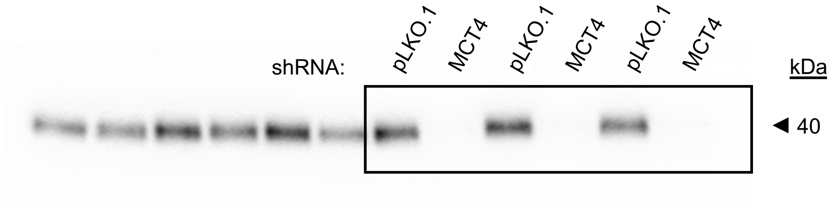

Unexposed (white light)

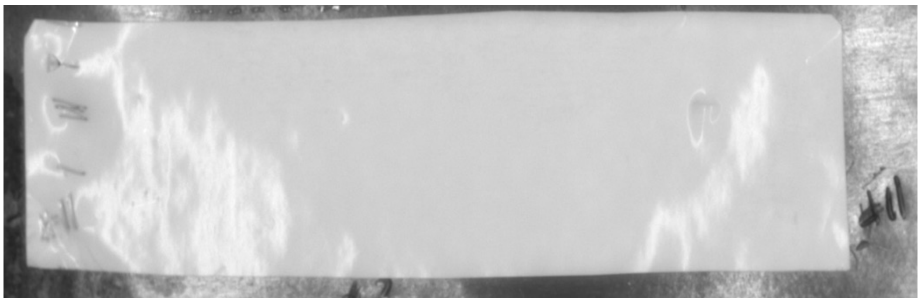

DCTN1 (MCT4)

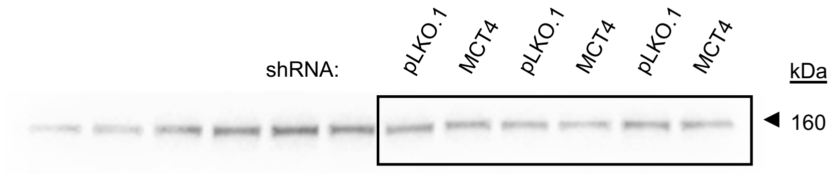

Unexposed (white light)

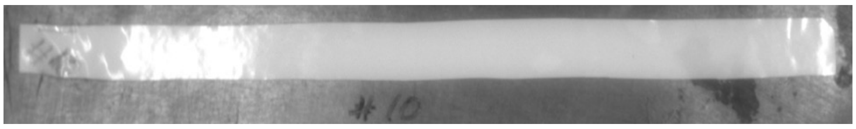

Journal of Cell Science • Supplementary information

Suppl. fig 3a continued

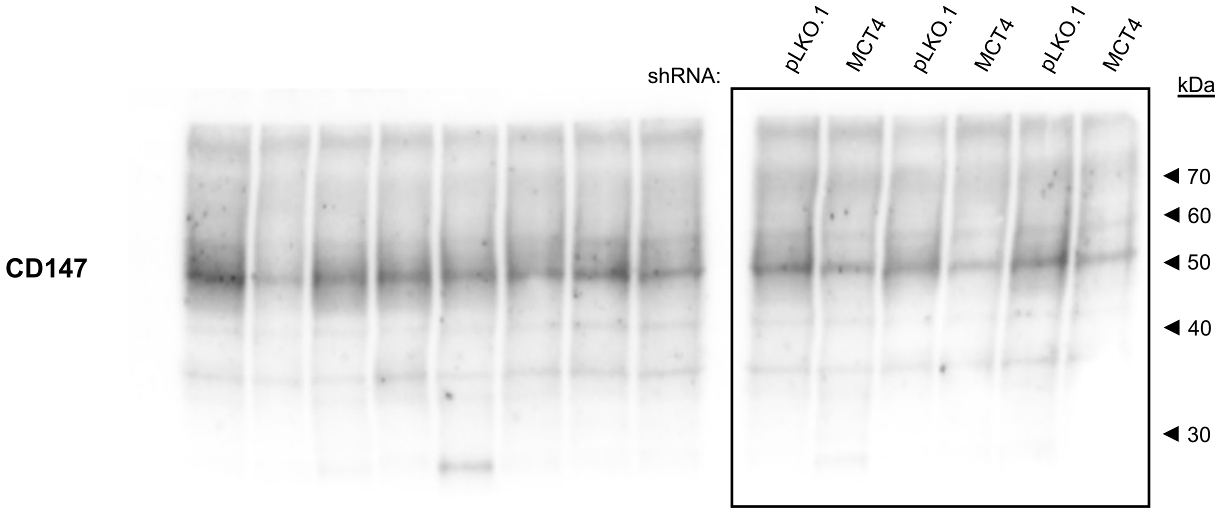

Unexposed (white light)

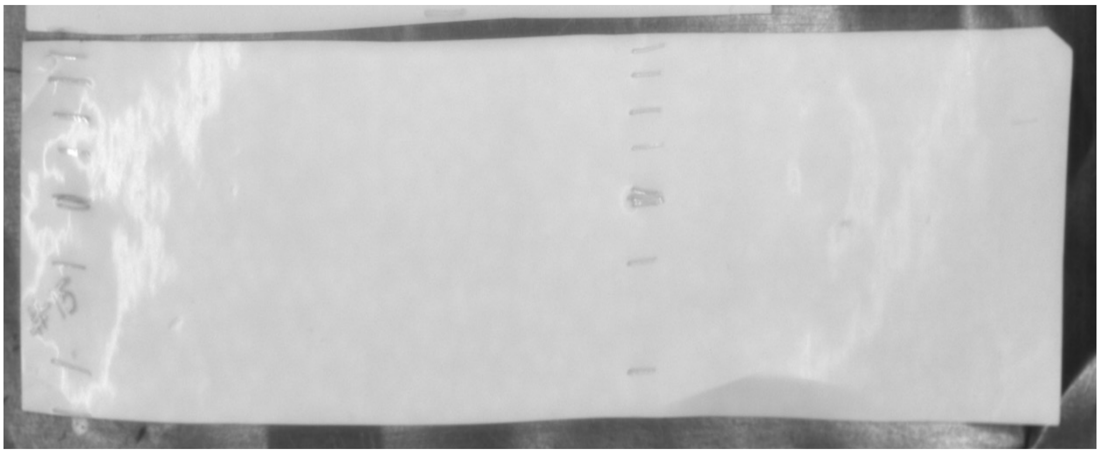

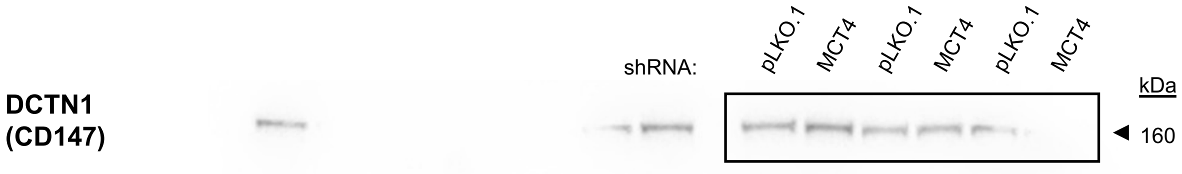

Unexposed (white light)

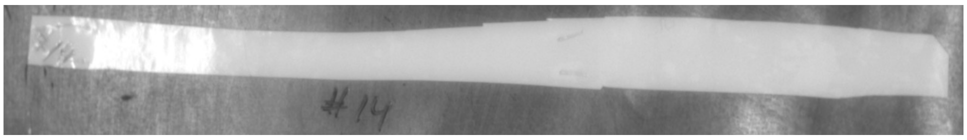

Fig. S7. Blot transparency

Raw data underlying the western blots shown.

Uncropped exposed membranes are shown for each figure. When data not included in the figure are present on the membrane (e.g. other experiments run in parallel), the parts used for the figures are outlined with a box. Available photos of the unexposed membranes (white light) are shown to the right of, or below, the exposed blot.

Journal of Cell Science • Supplementary information

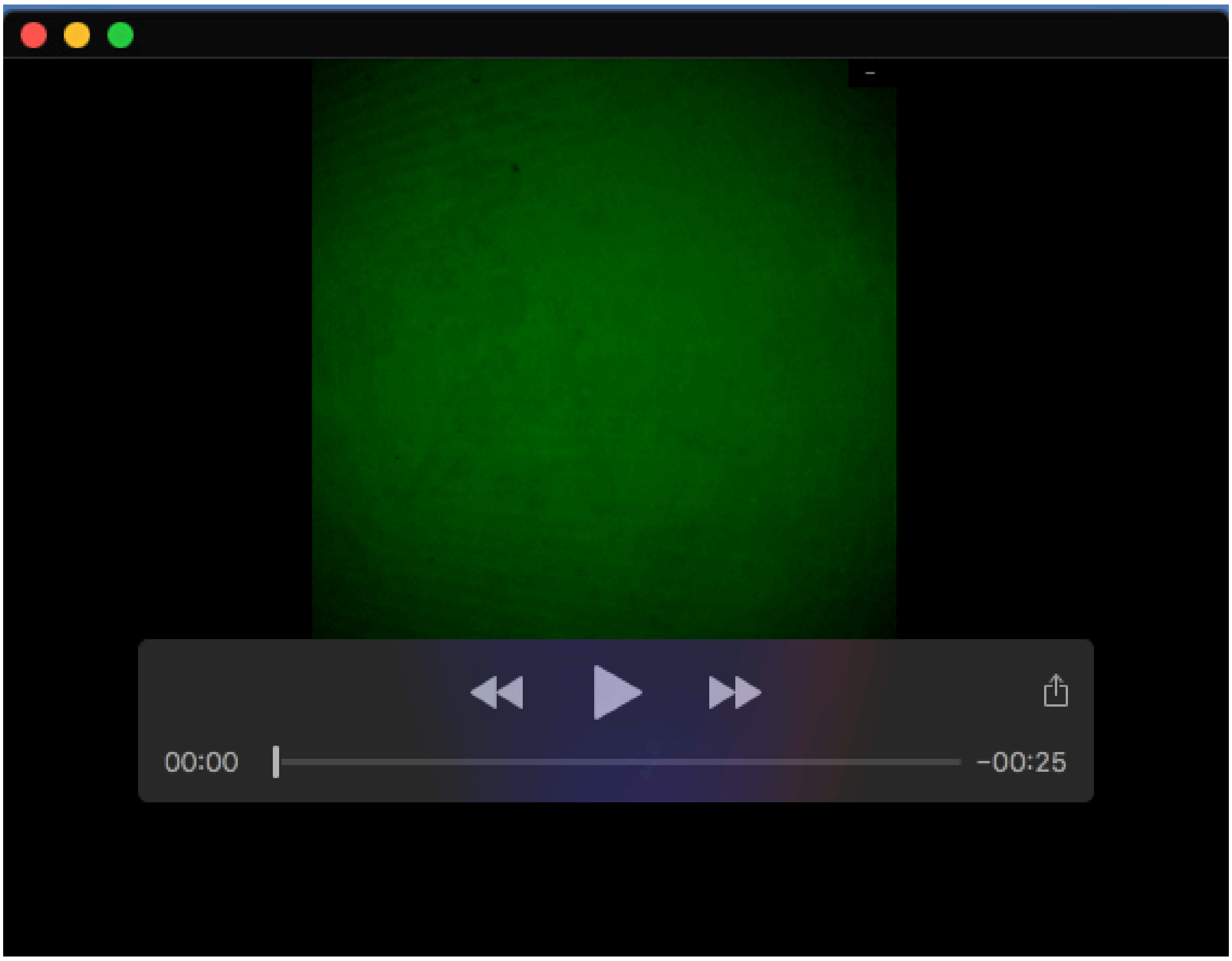

Movie 1. Live cell imaging of MDA-MB-231 cells growing on fluorescent gelatin.
WT MDA-MB-231 cells were seeded in an Oregon-green conjugated gelatin-coated μ-Slide 4-well plate and allowed to grow for 3 h in a CO2 incubator before being moved to the microscope stage. Images were acquired every 15 min for 14 h employing an Olympus IX83 microscope with a Yokogawa spinning disc, using brightfield illumination, the 488 confocal laser line, a 40X objective, and CellSens Dimension software. To enable live cell imaging, the microscope was set up to ensure proper conditions for cell culturing (5% CO2, 37°C, and connection to a humidifier).

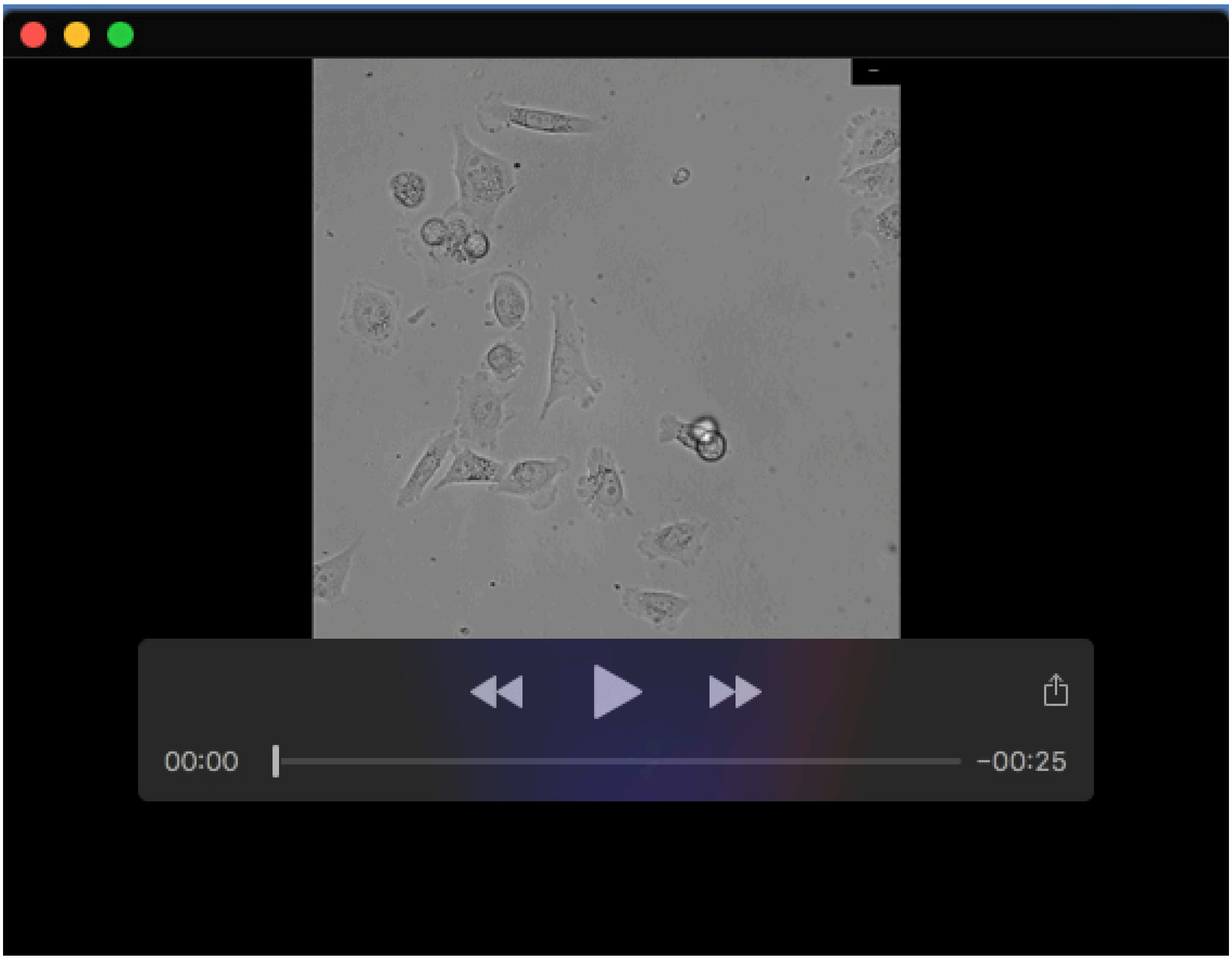

Movie 2. Live cell imaging of MDA-MB-231 cells growing on fluorescent gelatin.

WT MDA-MB-231 cells were seeded in an Oregon-green conjugated gelatin-coated μ-Slide 4-well plate and allowed to grow for 3 h in a CO2 incubator before being moved to the microscope stage. Images were acquired every 15 min for 14 h employing an Olympus IX83 microscope with a Yokogawa spinning disc, using brightfield illumination, the 488 confocal laser line, a 40X objective, and CellSens Dimension software. To enable live cell imaging, the microscope was set up to ensure proper conditions for cell culturing (5% CO2, 37°C, and connection to a humidifier).
