# Supplementary material for: MCT4 and CD147 colocalize with MMP14 in invadopodia and support matrix degradation and invasion by breast cancer cells
Source: J Cell Sci. 2024 Apr 30;137(8):jcs261608. doi: 10.1242/jcs.261608 (PMC11112124; doi:10.1242/jcs.261608)
Supplement: Supplementary file 1 [file joces-137-261608_review_history.pdf]

MCT4 and CD147 colocalize with MMP14 in invadopodia and support matrix degradation and invasion by breast cancer cells

Signe Meng, Ester E. Sørensen, Muthulakshmi Ponniah, Jeppe Thorlacius-Ussing, Roxane Crouigneau, Tanja Larsen, Magnus T. Borre, Nicholas Willumsen, Mette Flinck and Stine F. Pedersen

DOI: 10.1242/jcs.261608

Editor: Andrew Ewald

Review timeline

Original submission: 9 September 2023
Editorial decision: 30 October 2023
First revision received: 20 February 2024
Accepted: 23 March 2024

Original submission

First decision letter

MS ID#: JOCES/2023/261608

MS TITLE: MCT4 and CD147 co-localize with MMP14 in invadopodia and autolysosomes and collectively stimulate breast cancer cell invasion by increasing extracellular matrix degradation

AUTHORS: Signe Meng, Ester E Sorensen, Muthulakshmi Ponniah, Jeppe Thorlacius-Ussing, Roxane Crouigneau, Magnus T. Borre, Nicholas Willumsen, Mette Flinck, and Stine Falsig Pedersen

ARTICLE TYPE: Research Article

We have now reached a decision on the above manuscript.

To see the reviewers' reports and a copy of this decision letter, please go to: https://submit-jcs.biologists.org and click on the 'Manuscripts with Decisions' queue in the Author Area. (Corresponding author only has access to reviews.)

As you will see, the reviewers share enthusiasm for the study and raise a number of substantial criticisms that prevent me from accepting the paper at this stage. They suggest, however, that a revised version might prove acceptable, if you can address their concerns. Please let me know if you would like to prepare a revision plan for discussion; this is completely optional. If you think that you can deal satisfactorily with the criticisms on revision, I would be pleased to see a revised manuscript. We would then return it to the reviewers.

Please ensure that you clearly highlight all changes made in the revised manuscript. Please avoid using 'Tracked changes' in Word files as these are lost in PDF conversion.

I should be grateful if you would also provide a point-by-point response detailing how you have dealt with the points raised by the reviewers in the 'Response to Reviewers' box. Please attend to all of the reviewers' comments. If you do not agree with any of their criticisms or suggestions please explain clearly why this is so.

Reviewer 1

© 2024. Published by The Company of Biologists under the terms of the Creative Commons Attribution License (https://creativecommons.org/licenses/by/4.0/).

Advance summary and potential significance to field

The authors study the relationship between MCT4 and CD147, a transporter and its accessory subunit. This pair has been described in the past but the manner in which MCT4 or CD147 are essential individually or dually on ensemble functions such as cancer related MMP activation have been unclear. This study set to test these hypotheses and did so in a diligent manner. The assays using gels are elegant and informative. The paper makes a case for the proteins acting together to promote invasive behviour.

Comments for the author

The degradation assay is elegant and intuitive to interpret. To what degree could the degradation be due to acidification? This has not quite been shown because a suitable experiment would manipulate transport activiy by MCTs. Have the authors considered an experiment using galactose instead of glucose to reduce/suppress lactic acid production? Are there good MCT4 inhibitors? Alternatively, buffering capacity of the gel may be increased. Thes experiments would provide more compelling evidence that the collaboration causes invasive behaviour throguh acidication.

The conclusion that MCT OE increase lactate extrusion is not supported:

- The interpretation of Fig 2I is unclear. There are multiple factors that will determine lactate concentration, including cell number and metabolic rate aside from MCT mediated permeability. This particular experiment is therefore inconclusive and should either be removed or supplemented with additional data such as measurements of glucose consumption.
- Fig 2K data appear to show a trend but the experiment is underpowered and not well analysed – the traces should be calibrated and some measure of initial rate calculated, given the authrs talk about a rate. Either this data should be removed or supplemented with more analysis and measurements.

The effects on migration are very compelling. KD of CD147 was with two constructs, but MCT with one only- is there a reason why only one construct was used? A second construct would be desirable.

Minor
Intro – second paragraph. Since MCT is electroneutral, the relevant gradients will be chemical, rather than electrochemical.
Fig2 – x axis needs units.

<u>Reviewer 2</u>

Advance summary and potential significance to field

see attached

Comments for the author

Meng et al., report that the lactate transporter MCT4 and the chaperone CD147 favor breast cancer invasion through interdependent effects on ECM degradation. Their data are strong for showing an interdependence of MCT4 and CD147 for their expression and promoting invasion/ECM degradation, although interdependence of their expression is not entirely new. Showing co-localization in intracellular vesicles, suggested autolysosomes, is new, however, as described in the comments there are discrepancies for correlating effects on invasion compared with matrix degradation. Most important, although they claim new insight on mechanisms, this is rather limited and how a lactate transporter (or possibly H+ extrusion) is linked to MMP activity, matrix degradation, and invasion remains unresolved.

1. Suppl F1. Including expression levels in non-invasive MCF7 cells is informative; however, quantification from several cell preparations and blots, like in Fig. 1b, should be included. This is important, particularly for MCF7 cells because although MCF4 expression in vector

© 2024. Published by The Company of Biologists under the terms of the Creative Commons Attribution License (https://creativecommons.org/licenses/by/4.0/).

MDA-MB-231 cells is greater, the DCNT1 loading control suggests more protein. Also, it is unclear why 2 sets of MDA-MB-231 are shown.

2. Fig. 2. It is unclear how to interpret the significance of increased plasma membrane localization relative to what is emphasized from data in Fig. 6 and the schematic that an intracellular localization enables invasion. Additionally, in 2a the first column is not labeled and unclear. In 2f data on pHi is not linked to any sort of mechanism, and moreover absolute pHi (calibrated) is not shown but rather only SNARF ratios, which is not valid.

3. Fig. 3. These data are strong for correlating expression levels and invasion. It would be even more informative to integrate with data in Fig. S1. Does overexpression in noninvasive MCF- 7 cells increase invasion? Same for degradation as in shown in Fig. 4.

4. Fig. 4. Physiological relevance is improved by showing degradation of collagen and not just gelatin. However, it is unclear how to interpret some of these data on their own and also relative to those in Fig. 3. For a “big picture” interpretation, with KD of either, lack of a difference in degradation at 12 h compared with vector is a concern. Additionally, these time- dependent differences make it difficult to correlate with migration and invasion in Fig. 3, which were scored at 24 h. Also relative to Fig. 3, how does one interpret that overexpression of both proteins increases invasion but not matrix degradation, that overexpression of MCT4 increases invasion but not ECM degradation or MMP activity (reC1M index), and that overexpression of CD147 alone doesn’t increase invasion or ECM degradation but does increase MMP activity. These discrepancies make interpretation difficult, are not well explained, and do not support their conclusion (p13 Discussion) that *MCT4 and CD147 favor breast cancer cell invasiveness through interdependent effects on ECM degradation.*

5. Fig. 6. The significance of co-localization with LAMP1 is unclear, although cited by a previous article. How this links with a story on invasion is also unclear, because OE has effects on invasion and matrix degradation but is not scored relative to intravesical localization. These is there is evidence of autophagosome fusion with the plasma membrane and “release” as has previously suggested for how lysosomes enable cancer cell metastasis, but this is not shown.

6. Overall, the data are mostly descriptive with limited insight on mechanisms. Is MCT4 activity important vs the protein co-localizing with CD147? Does a wild type but not mutant inactive MCF4 restore invasion/matrix degradation with silencing? If matrix degradation is mediated by H+ efflux of MCT, why does matrix degradation increase with overexpression of CD147 alone but not MCT4 alone. An additional limitation is discrepancies of data in Fig. 3 and 4, as noted in comment #4.

Reviewer 3

Advance summary and potential significance to field

This is a well-crafted manuscript that shows that MCT4 and the CD147 chaperone regulate each other’s expression, colocalize both at the plasma membrane and in intracellular structures such as lysosomes, F-actin decorated large vesicles and partly in autophagosomes. Furthermore, this study shows that MCT4and CD147 are involved in cell invasion through their presence in invadopodes, as well as their ability to enhance matrix degradation by MMPs through H+ extrusion. This leads the authors to propose a model where MCT4-CD147 and MMP14 are endocytosed from the plasma membrane to be transferred to invadopodia. This work uses a wide variety of techniques to manipulate the expression of the studies proteins, as well as cell imaging; cytometry, pH measurements, motility and/or invasion assays.
Taken together this constitutes an solid study that leads to an interesting mechanism to explain the aggressivity of MCT4 and CD147 coexpression in tumor cells. I nevertheless have several points and questions that are listed thereafter.

© 2024. Published by The Company of Biologists under the terms of the Creative Commons Attribution License (https://creativecommons.org/licenses/by/4.0/).

Comments for the author

Main Points:
Point 1: The overexpression experiments are performed using transient transfection. So several experiments (motility assays, pH measurements upon lactate addition, lactate dosage…) show the average of the contribution of transfected and untransfected cells. Consequently, some of the reported effects could be affected by the transfection efficiency and possibly underestimated. Could it be possible to have an estimate of the % of transfected cells in these experiments?

Point 2: Do the overexpression or silencing of MCT4 or/and CD147 impact cell shape and the morphology/distribution of intracellular compartments? this is an important point that is very difficult to judge from the given images.

Point 3: This manuscript shows colocalization of MCT4 and CD147 but do they interact physically or are they just targeted to the same cellular localization? Could it be possible to perform crosslinking or CoIP to check this?

Point 4: The experiments that show that manipulating the expression of MCT4 at the plasma membrane can affect cells invasion suggest that it is the transport rather than lactate intracellular concentration itself that is limiting for proton extrusion. Could the authors therefore provide the reader with a status on the metabolism of the MDA-MB8231 in their culture and experimental conditions ?

Point 5: Pertaining to the previous point, adding 20 mM of extracellular lactate (close to the transporters' Km value) produces an intracellular acidification showing an inward lactate gradient. Could it be possible to estimate intracellular lactate concentrations in the different cells used in the study ?

Point 6: The authors could better quantify the pH changes of Figure 2k by fitting the points with exponentials (that globally reflect the changes of the probe protonation-deprotonation equilibrium, assuming that other mechanisms affecting pH are the same between the different cell lines) and compare their time constants instead of using initial rates that only take a few points into accounts.

Additional minor points:

Page 12 : "Furthermore, MMP14 is known to play a critical role in collagen-I phagocytosis and (Lee et al., 2006)". Something is missing in the sentence

---

First revision

Author response to reviewers' comments

PLEASE NOTE THAT THE RESPONSE LETTER CONTAINS FIGURES WHICH ARE LOST IN THIS VERSION - A PDF VERSION WITH THE FIGURES IS UPLOADED AS SUPPLEMENTARY INFORMATION, AS IS A TRACKED CHANGES VERSION OF THE MANUSCRIPT.

Point by point response to reviewers for the manuscript: "*MCT4 and CD147 co-localize with MMP14 in invadopodia and autolysosomes and collectively stimulate breast cancer cell invasion by increasing extracellular matrix degradation*"

Reviewer 1

The authors study the relationship between MCT4 and CD147, a transporter and its accessory subunit. This pair has been described in the past but the manner in which MCT4 or CD147 are essential individually or dually on ensemble functions such as cancer related MMP activation

© 2024. Published by The Company of Biologists under the terms of the Creative Commons Attribution License (https://creativecommons.org/licenses/by/4.0/).

have been unclear. This study set to test these hypotheses and did so in a diligent manner. The assays using gels are elegant and informative. The paper makes a case for the proteins acting together to promote invasive behviour.

Thank you for your positive remarks about our work!

Reviewer 1 Comments for the Author:

Comment 1
The degradation assay is elegant and intuitive to interpret. To what degree could the degradation be due to acidification? This has not quite been shown because a suitable experiment would manipulate transport activiy by MCTs. Have the authors considered an experiment using galactose instead of glucose to reduce/suppress lactic acid production? Are there good MCT4 inhibitors? Alternatively, buffering capacity of the gel may be increased. Thes experiments would provide more compelling evidence that the collaboration causes invasive behaviour throguh acidication.

Answer to comment 1
Thank you for this insightful comment. We agree that this would be very nice to directly demonstrate. However, as the reviewer points out, it is quite difficult to disentangle the acidosis from the lactate in these experiments. For instance, the introduction of galactose instead of glucose would cause a switch from glycolysis to oxidative phosphorylation, however, not only would this alter the state of the cells fundamentally in so many ways that the result would be difficult to interpret (including changes in cell number, as you also point out in Comment 2), but OXPHOS also produces acid, just in the form of CO_2 (for a recent review detailing this, please see Swietach, Boedtkjer and Pedersen 2023, Nat Rev Cancer 23(12) 825-41).

With respect to the MCT4 inhibitors, we agree that it would be interesting to supplement the knockdown and knockout experiments with pharmacological inhibition to potentially gain insight into whether the role of MCT4 is related to its function as a transporter or to its presence per se in the cells. Unfortunately, the commercially available MCT inhibitors are either specific to MCT1 or very unspecific, hence we cannot currently address this question in a reliable manner.

We have therefore instead emphasized this limitation of the study in the discussion, on p. 16 (last paragraph before the conclusion), where we write:

> "This opens several new questions, not answered in the present work. Thus, whether theroles of MCT4 and CD147 in ECM degradation involves a role in the large vesicles/LAVs and/or MCT4 activity in invadopodia, where increased lactate-H^+ efflux by MCT4 would create a local, pericellular acidic zone enhancing protease activity, remains to be determined. If MCT4 plays a role in the LAVs *per se*, it might contribute to their particularly acidic luminal pH and thus to intracellular, cathepsin- and MMP-mediated ECM degradation (Montcourrier et al., 1994, Montcourrier et al., 1990)."

Comment 2
The conclusion that MCT OE increase lactate extrusion is not supported: The interpretation of Fig 2I is unclear. There are multiple factors that will determine lactate concentration, including cell number and metabolic rate, aside from MCT mediated permeability. This particular experiment is therefore inconclusive and should either be removed or supplemented with additional data, such as measurements of glucose consumption.
Fig 2K data appear to show a trend but the experiment is underpowered and not well analysed - the traces should be calibrated and some measure of initial rate calculated, given the authrs talk about a rate. Either this data should be removed or supplemented with more analysis and measurements.

Answer to comment 2
We agree with the reviewer that the interpretation of Fig. 2I and 2K is not well supported, and that in particular the data in Fig. 2I might have other interpretations than an increase in MCT4 activity. As these data are not strictly necessary for our interpretation, we have opted to

© 2024. Published by The Company of Biologists under the terms of the Creative Commons Attribution License (https://creativecommons.org/licenses/by/4.0/).

remove these panels entirely from the figure. The text on p. 8 (Results) and on p. 14 (Discussion) has been revised accordingly. Thank you for pointing this out.

Comment 3
The effects on migration are very compelling. KD of CD147 was with two constructs, but MCT with one only- is there a reason why only one construct was used? A second construct would be desirable.

Answer to comment 3
Thank you, yes these data are quite clear. The reason that we only used one siRNA construct for MCT4 is that we instead supplemented with stable lentiviral shRNA mediated knockdown (KD) cell line for this protein. The data is shown on the right side of panel b and c in Fig. 3, and fully support the transient KD data.
The cell line was generated in a previous study from our group (Andersen et al 2018 Int J Cancer 142(12):2529-
42). The efficient KD of MCT4 and corresponding reduction in CD147 protein level in this cell line are shown in Suppl. Fig. 3a-c.

Minor
Intro – second paragraph. Since MCT is electroneutral, the relevant gradients will be chemical, rather than electrochemical.

Thank you – this has been corrected.

Fig2 – x axis needs units.

Thank you – we assume that you referred to Fig. 2b, which actually had the units in the bottom sub-panel, as all panels are the same. However, for clarity we have now added the x-axis labels to all sub-panels.

Reviewer 2 Comments for the Author:

Meng et al., report that the lactate transporter MCT4 and the chaperone CD147 favor breast cancer invasion through interdependent effects on ECM degradation. Their data are strong for showing an interdependence of MCT4 and CD147 for their expression and promoting invasion/ECM degradation, although interdependence of their expression is not entirely new. Showing co-localization in intracellular vesicles, suggested autolysosomes, is new, however, as described in the comments there are discrepancies for correlating effects on invasion compared with matrix degradation. Most important, although they claim new insight on mechanisms, this is rather limited and how a lactate transporter (or possibly H+ extrusion) is linked to MMP activity, matrix degradation, and invasion remains unresolved.

Comment 1
Suppl F1. Including expression levels in non-invasive MCF7 cells is informative; however, quantification from several cell preparations and blots, like in Fig. 1b, should be included. This is important, particularly for MCF7 cells because although MCF4 expression in vector MDA-MB-231 cells is greater, the DCNT1 loading control suggests more protein. Also, it is unclear why 2 sets of MDA-MB-231 are shown.

Answer to comment 1
Thank you for this comment. MCF7 cells are extremely different from MDA-MB-231 cells in numerous other ways than being non-invasive (for instance, they are of the luminal B subtype whereas MDA-MB-231 are triple- negative, they are metabolically fundamentally different, and very importantly, they express both MCT1 and MCT4, whereas MDA-MB-231 cells only express MCT4). We have therefore chosen to remove the comparison in the revised manuscript, and now focus on the quantified MCF10A cell data in this figure (which is now Suppl. Fig. 2 because of inclusion of an additional supplemental figure at the request of another reviewer). The corresponding text on p. 7 has been revised accordingly.

Comment 2

© 2024. Published by The Company of Biologists under the terms of the Creative Commons Attribution License (https://creativecommons.org/licenses/by/4.0/).

Fig. 2. It is unclear how to interpret the significance of increased plasma membrane localization relative to what is emphasized from data in Fig. 6 and the schematic that an intracellular localization enables invasion. Additionally, in 2a the first column is not labeled and unclear. In 2f data on pHi is not linked to any sort of mechanism, and moreover absolute pHi (calibrated) is not shown but rather only SNARF ratios, which is not valid.

Answer to comment 2
2.1 We thank you for pointing this out. We cannot say from our data that the MCT4-CD147-dependent matrix degradation happens only extracellularly, and we did not intend to imply that. However, you are completely right that one could get that impression from the schematic in Figure 6. We have revised the figure to show that degradation might also occur intracellularly in acidic vesicles, and this point has now been specified in the discussion on p. 16:

> "If MCT4 plays a role in the LAVs *per se*, it might contribute to their particularly acidic luminal pH and thus to intracellular, cathepsin- and MMP-mediated ECM degradation (Montcourrier et al., 1994, Montcourrier et al., 1990)."

2.2 Thank you for pointing out the lacking labeling of the first column in panel a. This has now been added.

2.3 SNARF ratios are in fact frequently and validly used on their own, depending on the question asked. In this case the use of an uncalibrated ratio was justified as the aim of the experiment was not to study absolute pH values but to use the relative changes as a readout of lactate transport. However, to accommodate the reviewer we have removed panel 2f, which was not necessary for our conclusions. The text on p. 8 has been revised accordingly.

Comment 3
Fig. 3. These data are strong for correlating expression levels and invasion. It would be even more informative to integrate with data in Fig. S1. Does overexpression in noninvasive MCF-7 cells increase invasion? Same for degradation as in shown in Fig. 4.

Answer to comment 3
As discussed in our answer to comment 1, this comparison is not informative because MCF7 cells are extremely different from MDA-MB-231 cells in numerous other ways than being non-invasive. We therefore found it more informative to perform both knockdown- and overexpression experiments in the same cell line, i.e. MDA-MB-231, and could show that knockdown of the two proteins decreases, and overexpression increases, invasiveness. It may be noted however, that previous work from others has shown that overexpression of CD147 – which already express MCT4 – increases invasiveness and invadopodia formation in non-cancer MCF10A cells (Grass et al 2012 J Cell Sci 125(3):777-788). Although we already cited this study, this information has now been added on p. 6. Thank you for bringing this up.

Comment 4
Fig. 4. Physiological relevance is improved by showing degradation of collagen and not just gelatin. However, it is unclear how to interpret some of these data on their own and also relative to those in Fig. 3. For a "big picture" interpretation, with KD of either, lack of a difference in degradation at 12 h compared with vector is a concern. Additionally, these timedependent differences make it difficult to correlate with migration and invasion in Fig. 3, which were scored at 24 h. Also relative to Fig. 3, how does one interpret that overexpression of both proteins increases invasion but not matrix degradation, that overexpression of MCT4 increases invasion but not ECM degradation or MMP activity (reC1M index), and that overexpression of CD147 alone doesn't increase invasion or ECM degradation but does increase MMP activity. These discrepancies make interpretation difficult, are not well explained, and do not support their conclusion (p13 Discussion) that MCT4 and CD147 favor breast cancer cell invasiveness through interdependent effects on ECM degradation.

Answer to comment 4
We apologize for not being sufficiently clear. There are two main questions in this comment by the reviewer, answered individually below:

© 2024. Published by The Company of Biologists under the terms of the Creative Commons Attribution License (https://creativecommons.org/licenses/by/4.0/).

4.1 *“lack of a difference in degradation at 12 h compared with vector is a concern.”*
Answer:
There are two reasons for the lack of significance at time 12 h:
(i) The variation in these difficult and time-consuming assays is substantial, and there is a clear and significant difference at time 8 h. At 12 h there is still a clear trend for less degradation after MCT4 or CD147 KD, and especially in the MCT4 KD condition it is evident that one experiment with a higher degree of matrix degradation at 12 h is responsible for the lack of statistical significance at this time.
(ii) A reduced difference in matrix degradation with time is to be expected in these assays, because the fluorescent gelatin is being used up locally, saturating the assay. This is evident from the substantial and increasingly large “black spots” in the images, clearly visible at 12 h. We cannot prevent this, given the random movement of the cells.
Collectively, therefore, and in combination with the overexpression- and reC1M data (see below), we think the data show a clear picture of matrix degradation being correlated with the expression of MCT4 and CD147.

4.2. *“how does one interpret that overexpression of both proteins increases invasion but not matrix degradation, that overexpression of MCT4 increases invasion but not ECM degradation or MMP activity (reC1M index), and that overexpression of CD147 alone doesn’t increase invasion or ECM degradation but does increase MMP activity”*

Answer:
(i) The invasion, gelatin degradation, and reC1M measurements are three completely different assay types, and therefore it is not meaningful to directly compare the time courses. Overexpression of both proteins does very strongly tend to increase gelatin degradation, but because of the large variance in the assay, this is not statistically significant. The collagen-I degradation measurements (Fig. 4g, reC1M level) have lower variance and provides the statistical significance.
(ii) Yes, it is notable that the impact of MCT4 overexpression on invasion seems to be greater than that on gelatin- and collagen-I degradation. It remains very possible that MCT4 has effects in this process beyond its role in matrix degradation, such as its known interaction with integrins, or MCT4-activity-driven local changes in cytosolic pH, which could regulate cytoskeletal organization, as well described for other pH regulatory proteins.
(iii) A probable reason for this is that the collagen-I degradation by MMPs (reC1M level) is not alone sufficient to elicit invasion in the more complex Matrigel-coated transwells but requires a broader matrix degradation that is dependent on both proteins.

We have now updated the text in the Discussion (p. 16) according to the answers provided as 4.1 and 4.2, and we think it has been substantially improved. We thank the reviewer for this helpful comment.

<u>Comment 5</u>
Fig. 6. The significance of co-localization with LAMP1 is unclear, although cited by a previous article. How this links with a story on invasion is also unclear, because OE has effects oninvasion and matrix degradation but is not scored relative to intravesical localization. These is there is evidence of autophagosome fusion with the plasma membrane and “release” as has previously suggested for how lysosomes enable cancer cell metastasis, but this is not shown.

<u>Answer to comment 5</u>
The comment is not fully legible, but if we understand it correctly, the reviewer refers to a model in which the fusion of autophagosomes with the plasma membrane could cause the release of intralysosomal proteases to the exterior, contributing to the matrix degradation. This is of course possible, but as the reviewer points out, we have no evidence of this process and do not think it is the explanation for our data. The evidence that we present is that there is co-localization of MMP14, MCT4 and CD147 in LAMP1-positive vesicles, and that these also sometimes contain fluorescent gelatin. We therefore suggest that part of the MCT4-CD147-dependent matrix degradation could occur within these vesicles. We cannot be sure of their exact identity, but we know that they are LAMP1-positive.

<u>Comment 6</u>

© 2024. Published by The Company of Biologists under the terms of the Creative Commons Attribution License (https://creativecommons.org/licenses/by/4.0/).

Overall, the data are mostly descriptive with limited insight on mechanisms. Is MCT4 activity important vs the protein co-localizing with CD147? Does a wild type but not mutant inactive MCF4 restore invasion/matrix degradation with silencing? If matrix degradation is mediated by H+ efflux of MCT, why does matrix degradation increase with overexpression of CD147 alone but not MCT4 alone. An additional limitation is discrepancies of data in Fig. 3 and 4, as noted in comment #4.

<u>Answer to comment 6</u>
Thank you for these questions. They are listed below and separate answers provided for each:

6.1 *Is MCT4 activity important vs the protein co-localizing with CD147?*
As stressed in the Discussion section (p 13, first paragraph of the Discussion), activity and co-localization cannot be separated, because MCT4 is only functional when localized to the correct membrane, and this requires its interaction with CD147. To further emphasize the close interaction of the two, we have now included additional experiments (Suppl. Fig. 2e-g, text on p. 7 and 14) showing that MCT4 and CD147 co- immunoprecipitate (as previously shown by others), confirming their physical interaction.

6.2 *Does a wild type but not mutant inactive MCF4 restore invasion/matrix degradation with silencing?* Previous work has shown that the role of MCT4 in cancer cell invasion is prevented by a point mutation rendering the transporter inactive (Li et al 2021 Oncol. Lett 21(1):44, doi: 10.3892/ol.2020.12305). This is consistent with our previous work showing that both inhibition and knockdown of MCTs limits migration and invasion of cancer cells (e.g. Andersen et al 2018 Int J Cancer, doi: 10.1002/ijc.31276; Kong et al 2016 Pancreas, doi: 10.1097/MPA.0000000000000571). These papers have now been cited and the point emphasized further in the text, on p. 14. Thank you for alerting us to the paper using the inactivating point mutation.

6.3 *If matrix degradation is mediated by H+ efflux of MCT, why does matrix degradation increase with overexpression of CD147 alone but not MCT4 alone.*
We cannot with certainty explain why the increase in reCM1 level after MCT4 overexpression alone did not reach statistical significance. It is possible that CD147 levels were, in this set of experiments, rate-limiting for the process. However, it is also conceivable that this just reflects the greater variance of this data, that statistical significance was not reached. To avoid misunderstandings, we have now specified on p. 10 of the revised manuscript that the increase in reC1M levels after MCT4 expression alone did not reach statistical significance.

6.4 *An additional limitation is discrepancies of data in Fig. 3 and 4, as noted in comment #4.*
This has been answered in detail under comment 4, and as explained therein, we, respectfully, do not see these data as discrepancies but as differences reflecting the different assay types.

Reviewer 3
This is a well-crafted manuscript that shows that MCT4 and the CD147 chaperone regulate each other's expression, colocalize both at the plasma membrane and in intracellular structures such as lysosomes, F-actin decorated large vesicles and partly in autophagosomes. Furthermore, this study shows that MCT4and CD147 are involved in cell invasion through their presence in invadopodes, as well as their ability to enhance matrix degradation by MMPs through H+ extrusion. This leads the authors to propose a model where MCT4-CD147 and MMP14 are endocytosed from the plasma membrane to be transferred to invadopodia. This work uses a wide variety of techniques to manipulate the expression of the studies proteins, as well as cell imaging; cytometry, pH measurements, motility and/or invasion assays. Taken together this constitutes an solid study that leads to an interesting mechanism to explain the aggressivity of MCT4 and CD147 coexpression in tumor cells. I nevertheless have several points and questions that are listed thereafter.

Thank you very much for your clear understanding of the study and positive remarks about our work.

Reviewer 3 Comments for the Author:
Main Points:
<u>Point 1:</u>

© 2024. Published by The Company of Biologists under the terms of the Creative Commons Attribution License (https://creativecommons.org/licenses/by/4.0/).

The overexpression experiments are performed using transient transfection. So several experiments (motility assays, pH measurements upon lactate addition, lactate dosage…) show the average of the contribution of transfected and untransfected cells. Consequently, some of the reported effects could be affected by the transfection efficiency and possibly underestimated. Could it be possible to have an estimate of the % of transfected cells in these experiments?

Answer to Point 1:
Thank you for this very relevant comment. We agree that average effects are likely underestimated because of the contributions from untransfected cells. Because the untransfected cells also contain rather substantial amounts of endogenous MCT4 and CD147, the aim of the overexpression experiments was only to show that this enhances the effects, hence, we do not think the underestimation is problematic for the conclusions drawn. However, it is an excellent point, and we have now mentioned this in the text on p. 6, and provided the approximate transfection rates, based on 4 experiments, on p. 6 and in the Materials and Methods section, on p. 19. Because of the limit on the number of supplementary figures, we cannot include the calculations of transfection efficacy in the manuscript but have provided them here for your information (Fig. 1 for reviewer).

	MCT4			
	N	Picture	# OE cell	# cells total
IF#1	1	1	4	40
	1	2	0	20
	1	3	5	41
	1	4	1	26
IF#2	2	1	4	37
	2	2	3	46
	2	3	6	43
	2	4	8	56
IF#4	3	1	3	29
	3	2	2	31
	3	3	1	25
	3	4	2	31
	3	5	3	32
IF#5	4	1	3	24
	4	2	1	22
	4	3	3	33
	4	4	1	19
	4	5	2	36
	Total		52	591

	CD147			
	N	Picture	# OE cell	# cells total
IF#1	1	1	7	25
	1	2	6	28
	1	3	7	28
	1	4	3	27
	1	5	3	24
IF#2	2	1	4	23
	2	2	7	31
	2	3	7	37
	2	4	9	40
IF#4	3	1	3	31
	3	2	1	34
	3	3	2	30
	3	4	5	27
	3	5	4	35
	3	6	3	37
IF#5	4	1	2	31
	4	2	2	35
	4	3	3	31
	4	4	3	31
	4	5	1	28
	Total		82	613

	Transfection efficiency	
	MCT4	CD147
	8.8%	13.4%
Weighted average:		
N	MCT4	CD147
1	8%	20%
2	12%	21%
3	7%	9%
4	7%	7%
Average	8.6%	14.2%

Fig. 1 for reviewer. Overview of calculations of transfection efficiency. Cells with clearly increased MCT4 or CD147 expression were counted manually in 4-5 images per immunofluorescence analysis experiment. See text for explanation.

Point 2:
Do the overexpression or silencing of MCT4 or/and CD147 impact cell shape and the morphology/distribution of intracellular compartments? this is an important point that is very difficult to judge from the given images.

Answer to Point 2:
We fully agree that this is an important point. We carefully inspected our immunofluorescence analyses of MDA-MB-231 cells with overexpression or knockdown of the two proteins in cells seeded on gelatin-coated coverslips, using rhodamine-phalloidin staining to mark cell morphology. In our opinion there are no detectable changes in overall cell morphology. A supplementary figure documenting this has been included as new Suppl. Fig. 1, and the text on p. 6 has been expanded to indicate this point.

Point 3:
This manuscript shows colocalization of MCT4 and CD147 but do they interact physically or are they just targeted to the same cellular localization? Could it be possible to perform crosslinking or CoIP to check this?

Answer to Point 3:
Thank you for this comment. Yes MCT4 and CD147 physically interact. This has been shown previously in other studies, which is why we did not include a co-IP experiment in the first version of the manuscript. However, because of your comment, we realized that this experiment

© 2024. Published by The Company of Biologists under the terms of the Creative Commons Attribution License (https://creativecommons.org/licenses/by/4.0/).

was missing. We have therefore now performed co-IP experiments showing that MCT4 and CD147 reciprocally co-immunoprecipitate in MDA-MB-231 cells.
These experiments are shown as new Suppl. Fig. 2 e-g, and discussed in the manuscript text on p. 7 and 14. A new methods section has been included accordingly.

Point 4:
The experiments that show that manipulating the expression of MCT4 at the plasma membrane can affect cells invasion suggest that it is the transport rather than lactate intracellular concentration itself that is limiting for proton extrusion. Could the authors therefore provide the reader with a status on the metabolism of the MDA- MB8231 in their culture and experimental conditions?

Answer to Point 4:

Thank you for this point. We have previously demonstrated using Seahorse technology that under culture conditions similar to those used in the present study, MDA-MB-231 cells in which MCT4 levels are not manipulated are predominantly glycolytic (Rolver et al 2023 Int J Cancer, doi 10.1002/ijc.34404) consistent with other studies comparing this cell line to other breast cancer cell lines (Martin & McGee 2019 Cancer Metab, doi: 10.1186/s40170-019-0207-x). To gain insight into how MCT4 and CD147 manipulation might impact lactate homeostasis, we determined intracellular lactate levels after MCT4 and CD147 overexpression (Fig. 2 for reviewer). It may be noted that extracellular lactate levels in the culture medium were shown in the original manuscript as Fig. 1m, however, another reviewer requested these removed. As seen, overexpression of either MCT4 or CD147 increases intracellular lactate levels. While this might at first sight seem counterintuitive, we speculate that this reflects triggering of increased glycolytic activity by the increased availability of the lactate efflux pathway provided by MCT4. Consistent with this, also extracellular lactate levels were increased (see Fig. 1m of the original manuscript).

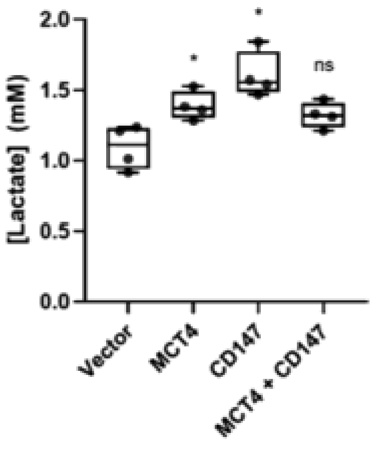

Fig. 2 for reviewer. Effect of overexpression of MCT4, CD147 or both, on intracellular lactate concentrations
MDA-MB-231 cells were transfected with empty pcDNA3.1 (+) vector as a negative control or MCT4, CD147 or MCT4 and CD147 plasmid constructs. 48 h post transfection, cells were then lysed and prepared for lactate measurements. Boxplot of mean intracellular lactate concentration, with each biological repeat shown as closed symbols, and median shown as black line. n=4. One-way ANOVA followed by Dunnett's multiple comparisons post-test was used to test for statistically significant differences ($p<0.05$) between treatment and control. * denotes $p<0.05$, ns: not significant.

Point 5:
Pertaining to the previous point, adding 20 mM of extracellular lactate (close to the transporters' Km value) produces an intracellular acidification showing an inward lactate gradient. Could it be possible to estimate intracellular lactate concentrations in the different cells used in the study ?

© 2024. Published by The Company of Biologists under the terms of the Creative Commons Attribution License (https://creativecommons.org/licenses/by/4.0/).

Answer to Point 5:
Yes indeed – these have been provided above (Fig. 2 for reviewer). As seen, overexpression of either MCT4 or CD147 increases intracellular lactate levels. As noted in the answer to Point 4, we think this most likely reflects an increased glycolytic activity triggered by the increased availability of the lactate efflux pathway provided by MCT4. Consistent with this, also extracellular lactate levels were increased under these conditions (Fig. 1m of the original manuscript, now removed as requested by another reviewer).

Point 6:
The authors could better quantify the pH changes of Figure 2k by fitting the points with exponentials (that globally reflect the changes of the probe protonation-deprotonation equilibrium, assuming that other mechanisms affecting pH are the same between the different cell lines) and compare their time constants instead of using initial rates that only take a few points into accounts.

Answer to Point 6:
Thank you for pointing this out. We agree – however, at the request of one of the other reviewers, this panel has been removed.

Additional minor points:

Page 12 : "Furthermore, MMP14 is known to play a critical role in collagen-I phagocytosis and (Lee et al., 2006)". Something is missing in the sentence.
Thank you very much for pointing this out. The "and" was removed to correct the sentence.

Second decision letter

MS ID#: JOCES/2023/261608

MS TITLE: MCT4 and CD147 co-localize with MMP14 in invadopodia and support matrix degradation and invasion by breast cancer cells

AUTHORS: Signe Meng, Ester E Sorensen, Muthulakshmi Ponniah, Jeppe Thorlacius-Ussing, Roxane Crouigneau, Tanja Larsen, Magnus T. Borre, Nicholas Willumsen, Mette Flinck, and Stine Falsig Pedersen

I am happy to tell you that your manuscript has been accepted for publication in Journal of Cell Science, pending standard ethics checks.

Reviewer 1

Advance summary and potential significance to field

All comments answered well and accurately

Comments for the author

No further comments

Reviewer 2

Advance summary and potential significance to field

© 2024. Published by The Company of Biologists under the terms of the Creative Commons Attribution License (https://creativecommons.org/licenses/by/4.0/).

The revision is mostly cosmetic without including any new data suggested by the previous review. The cosmetic edits do improve clarity but not requested mechanistic insights. However, as is the work provides new insights - albeit limited, and is worthy of publication

Comments for the author

Comments above

Reviewer 3

Advance summary and potential significance to field

In my opinion, the authors have made a great work and now submit a revised version with additional figures and text that now concurr to make and excellent study.

Comments for the author

no minor comments

© 2024. Published by The Company of Biologists under the terms of the Creative Commons Attribution License (https://creativecommons.org/licenses/by/4.0/).
